# Heterogeneity‐induced NGF‐NGFR communication inefficiency promotes mitotic spindle disorganization in exhausted T cells through PREX1 suppression to impair the anti‐tumor immunotherapy with PD‐1 mAb in hepatocellular carcinoma

**DOI:** 10.1002/cam4.6736

**Published:** 2024-01-10

**Authors:** Xin Wang, Tongwang Yang, Shangheng Shi, Chuanshen Xu, Feng Wang, Deshu Dai, Ge Guan, Yong Zhang, Shuxian Wang, Jianhong Wang, Bingliang Zhang, Peng Liu, Xiaoshuai Bai, Yan Jin, Xinqiang Li, Cunle Zhu, Dexi Chen, Qingguo Xu, Yuan Guo

**Affiliations:** ^1^ Liver Disease Center The Affiliated Hospital of Qingdao University Qingdao China; ^2^ Academician Workstation Changsha Medical University Changsha China; ^3^ Hunan Key Laboratory of the Research and Development of Novel Pharmaceutical Preparations Changsha Medical University Changsha China; ^4^ Beijing Institute of Hepatology Capital Medical University Beijing China

**Keywords:** hepatocellular carcinoma, immunotherapy, nerve growth factor, nerve growth factor receptor, pd‐1

## Abstract

**Background:**

The mechanism of decreased T cells infiltrating tumor tissues in hepatocellular carcinoma is poorly understood.

**Methods:**

Cells were separated from the single‐cell RNA‐sequence dataset of hepatocellular carcinoma patients (GSE149614) for cell‐cell communication. Flow cytometry, EDU staining, H3‐Ser28 staining, confocal immunofluorescence staining, western blotting and naked microsubcutaneous tumors were performed for the mechanism of NGF‐NGFR promoting proliferation.

**Results:**

The present study has revealed that during the process of T‐cell infiltration from adjacent tissues to tumor tissues, an inefficiency in NGF‐NGFR communication occurs in the tumor tissues. Importantly, NGF secreted by tumor cells interacts with NGFR present on the membranes of the infiltrated T cells, thereby promoting the proliferation through the activation of mitotic spindle signals. Mechanistically, the mediation of mitotic spindle signal activation promoting proliferation is executed by HDAC1‐mediated inhibition of unclear trans‐localization of PREX1. Furthermore, PD‐1 mAb acts synergistically with the NGF‐NGFR communication to suppress tumor progression in both mouse models and HCC patients. Additionally, NGF‐NGFR communication was positively correlates with the PD‐1/PDL‐1 expression. However, expressions of NGF and NGFR are low in tumor tissues, which is responsible for the invasive clinicopathological features and the disappointing prognosis in HCC patients.

**Conclusion:**

Inefficiency in NGF‐NGFR communication impairs PD‐1 mAb immunotherapy and could thus be utilized as a novel therapeutic target in the treatment of HCC patients in clinical practice.

## Graphical abstract

NGF secreted by tumor cells interacts with NGFR present on the membranes of the infiltrated T cells, thereby promoting the proliferation through the activation of mitotic spindle signals. The mediation of mitotic spindle signal activation promoting proliferation is executed by HDAC1‐mediated inhibition of unclear trans‐localization of PREX1.

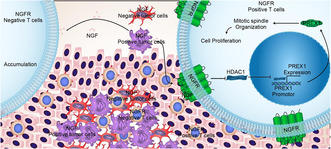

## 1 INTRODUCTION

In tumor tissues, the infiltrated T cells with reprogrammed gene expression profiles undergo an exhaustion process due to continuous stimulation from tumor antigens.^1^, ^2^ The exhausted T cells exhibit an impaired functional state with altered LAG‐3, BTLA, PD‐1, CD160, 2B4, CTLA‐4, and TIM‐3 expressions. In other words, T cells infiltrated in the tumor tissue are in a state of functional exhaustion.^3^, ^4^ Interestingly, a previous study by our research group demonstrated a dramatic decrease in the number of T cells in tumor tissue, indicating the state of quantitative exhaustion of these T cells.^5^ The existing literature on the quantitative exhaustion of these T cells mainly focuses on the regulation of apoptosis,^6^, ^7^, ^8^ while the functional role and the underlying molecular mechanism of the proliferation of T cells in the state of quantitative exhaustion remain largely unclear.

The nerve growth factor, which is the earliest identified member of the neurotrophic factor family, regulates biological functions by binding to the receptor present on the membrane of cells.^9^, ^10^ The receptors of the nerve growth factor are of two types: low‐affinity nerve growth factor (p75NTR, also referred to as NGFR) and high‐affinity tyrosine kinase receptor (TrkA).^11^ NGFR has a relatively simple structure and interacts non‐specificity with four neurotrophic factors, namely NT‐4, NT‐3, brain‐derived neurotrophic factor (BDNF), and nerve growth factor (NGF).^12^ NGFR performs a positive regulatory function in cell survival and proliferation in synergy with TrkA while promoting apoptosis in the absence of TrkA. The other three neurotrophic factors do not exhibit such positive regulatory roles in cell proliferation. The regulatory role of NGFR in multiple biological processes is known to be dependent on the cooperation of Trks.^13^, ^14^ However, the functional role of nerve growth factor receptors and other factors in the infiltrated T cells in hepatocellular carcinoma and the underlying molecular mechanisms have not been elucidated so far.

## 2 MATERIALS AND METHODS

### 2.1 Animals and patients

Tumor tissue and adjacent tissue samples from 98 HCC patients were collected for RNA and protein expression extraction from the Affiliated Hospital of Qingdao University between June 2018 and November 2021 (Table S1). Twenty‐nine PD‐1‐positive HCC patients, who received the PD‐1 mAb therapy in the Affiliated Hospital of Qingdao University between January 2019 and December 2021 were included as the study population (Table S2). Paraffin sections of the samples were evaluated for NGF and NGFR expressions using immunocytochemistry. Healthy donors from the Organ Transplantation Center at the Affiliated Hospital of Qingdao University were recruited for the collection of peripheral blood samples. The use of the tissue and blood samples in the present study was performed according to the Declaration of Helsinki. All individual participants or families were thoroughly informed regarding the study and were asked to sign a written informed consent form.

BALB/c nude mice purchased and authorized from SPF (Beijing) Biotechnology Co. Ltd. were used in the present study. The animals were maintained in cages in the Specific Pathogen Free house at the animal center of Qingdao University. The guidelines of the National Institutes of Health and the Qingdao University Animal Care Facility were followed in the animal project. The ethics committee of the Affiliated Hospital of Qingdao University approved the project (QYFYWZLL27133).

### 2.2 Data analysis

High‐quality cells were separated from GSE149614 based on the criteria percent.mt < 15, nCount_RNA > 1000, nCount_RNA < 20,000 and nFeature_RNA > 500. Human Cell Atlas Data were utilized to annotate cells under the function “FindClusters.” Gene expression profile datasets (GSE14520, GSE22058, GSE25097, GSE36376, GSE46444, GSE54236, GSE57957, GSE64041, and GSE76297) of HCC were collected directly from the GEO. Pan‐cancer gene expression datasets were downloaded from the TCGA (https://portal.gdc.cancer.gov). Two‐tailed Student's *t*‐tests were performed to analyze the differences in the variable genes. The values of overall, disease‐specific, disease‐free, and progression‐free survival were determined using Log‐rank tests. Multivariate analyses were performed under a stepwise Cox multivariate proportional hazard regression model. IBM SPSS statistic 22 was used for performing the statistical analyses. Variable genes were conducted between adjacent and tumor tissue. A *p*‐value of <0.05 was used as the threshold of statistical significance. Values were expressed as mean ± SD.

## 3 RESULTS

### 3.1 NGF‐NGFR communication inefficiency in tumor tissues of HCC patients

**FIGURE 1:**
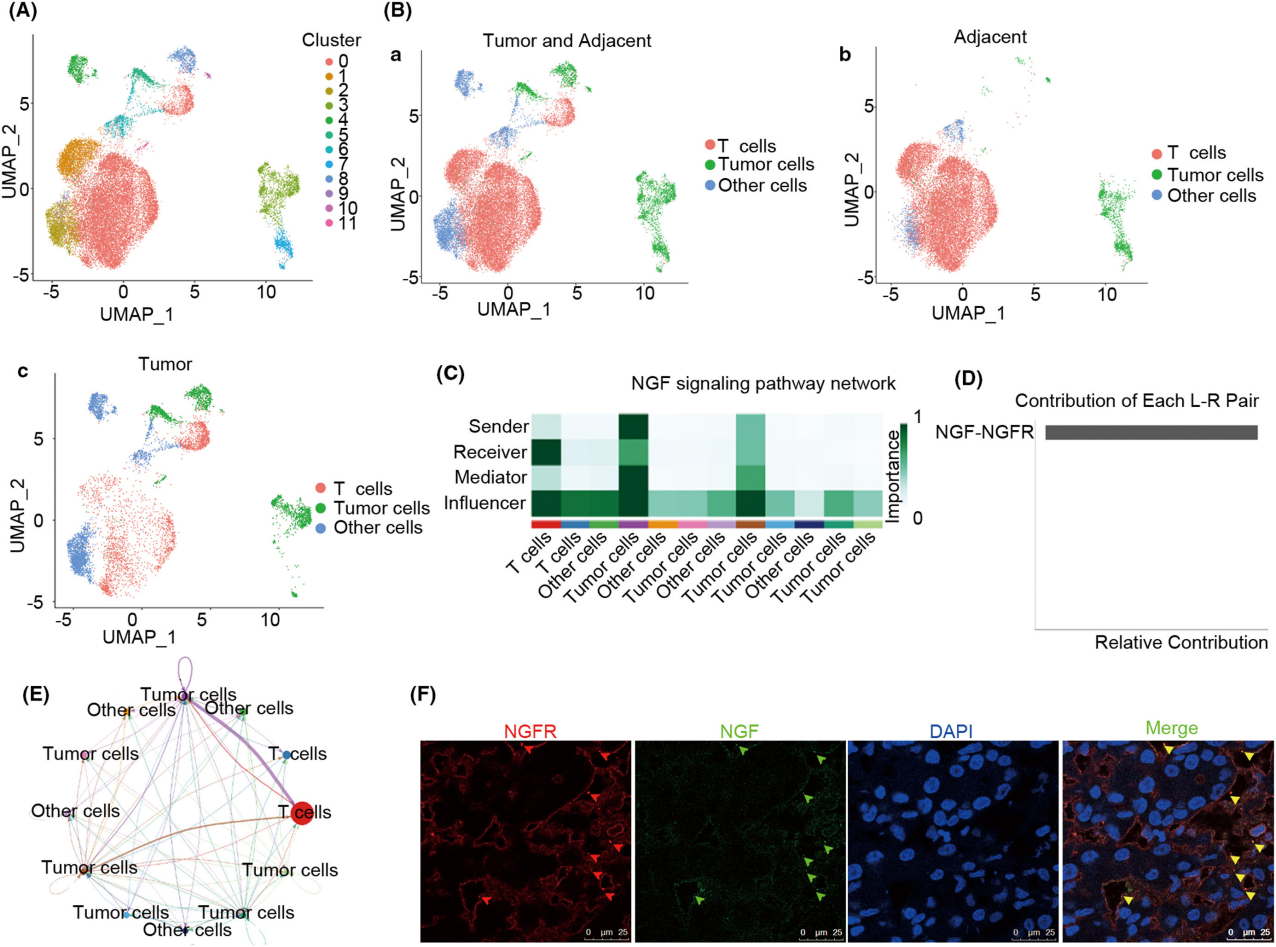
The cell–cell communication between tumor cells and T cells. (A) The UMAP reveals clusters of cells in HCC patients. (B) The UMAP reveals the tumor cells and T cells in HCC patients. (C) The NGF signaling pathway network between the tumor cells and T cells. Clusters in the NGF signaling pathways are depicted in color. (D) Ligands and receptors contribute to the NGF signaling pathway between the tumor cells and T cells. (E) The circle plot depicts the NGF signaling pathway between the tumor cells and T cells. Clusters in the NGF signaling pathways are depicted in color and line. (F) NGF (green) and NGFR (red) co‐colocalization in the HCC patients. DAPI was used for staining the genomic DNA (blue). Scale bar, 25 μm.

Although T cells are quantitatively exhausted in tumor tissues, the mechanism underlying this T‐cell exhaustion is poorly understood to date. In the present study, T cells and tumor cells were separated from the tumor tissue and adjacent tissue samples from the single‐cell RNA‐sequence dataset (Figure 1A, GSE149614).^15^ It was observed that few T cells had infiltrated into to tumor tissues of HCC patients (Figure 1B; Table S4). Subsequently, the CellChat R package was used for evaluating the results of the cell–cell communication assay. Importantly, the NGF secreted from tumor cells was received mainly by the NGFR expressed in T cells (Figure 1C), and this NGF‐NGFR interaction contributed to the primary NGF‐NGFR communication signaling pathway network (Figure 1D). Unfortunately, only two clusters of tumor cells (cluster 3 and cluster 7, which were from adjacent tumor tissues) successfully secreted NGF individually, while tumor cells in clusters 5, 8, 10, and 11 (tumor tissues) failed to secrete NGF (Figure 1E). Furthermore, the double immunofluorescence assay was performed to evaluate the NGF‐NGFR communication in vivo. NGF was observed to be co‐localized with NGFR on the membrane of the infiltrated T cells in the tumor tissues of HCC patients (Figure 1F). Together, these results suggested that the tumor cell‐secreted NGF was received specifically by the NGFR expressed in T cells, and NGF‐NGFR communication was inefficient in the tumor tissues of HCC patients.

### 3.2 The exhaustion of T cells in HCC patients was driven by the infiltration process

**FIGURE 2:**
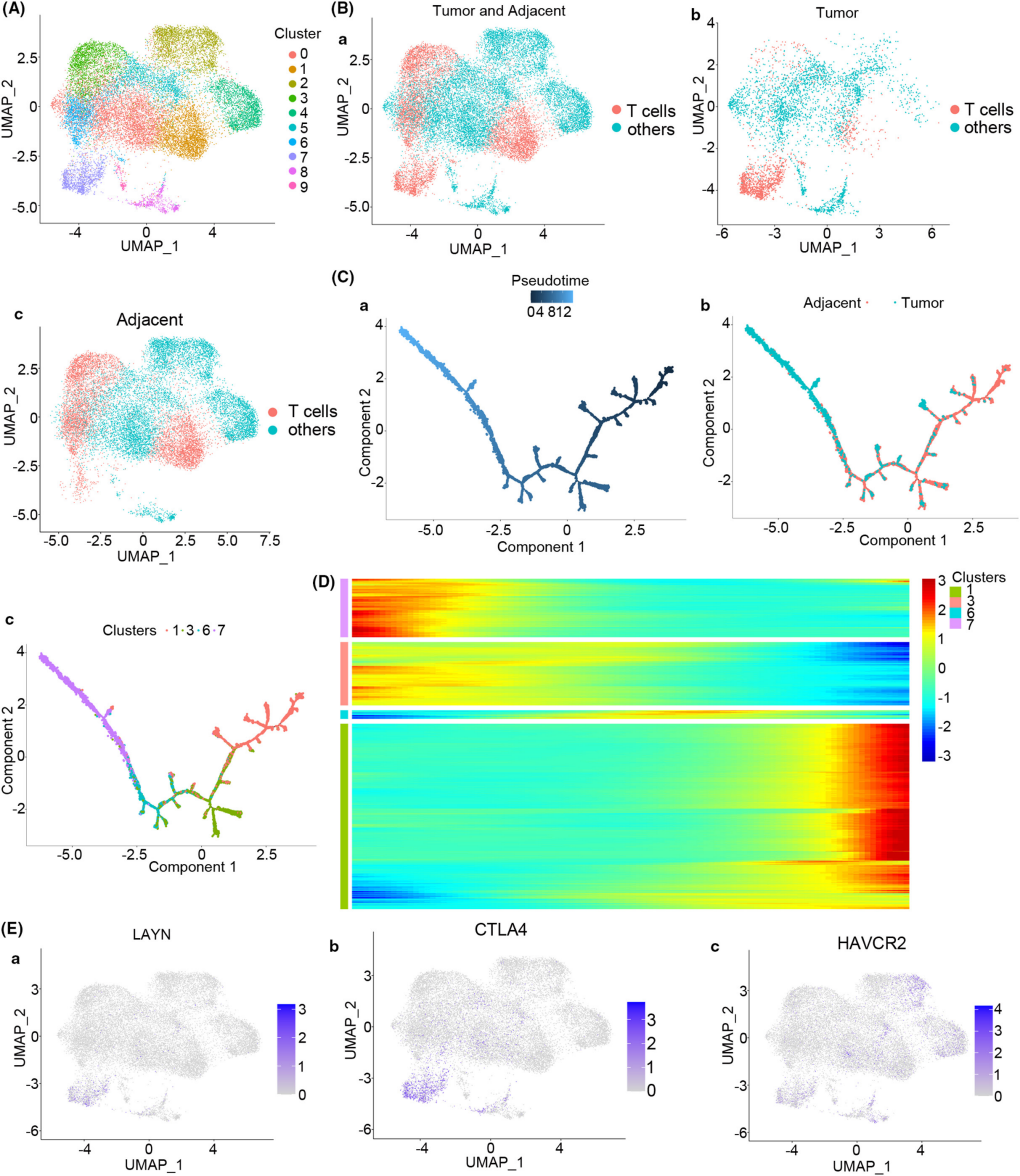
Diffusion pseudo‐time analysis of T cells in HCC patients. (A) The UMAP depicts clusters of T cells in the tumor tissues and adjacent tissues. (B) The UMAP depicts T cells in tumor and adjacent tissues (Ba), tumor tissues (Bb), and adjacent tissues (Bc) of HCC patients. (C) T cells were pseudo‐time differentiated with one main distinct branch point adjacent to the tumor tissues. Ca cell differentiation map for pseudo‐time, Cb cell differentiation map for tumor and adjacent tissues, and Cc cell differentiation map for cluster. (D) Marker genes (top 10) of the sub‐population that were reprogrammed in this pseudo‐time process. (E) The UMAP for the expression of the T‐cell exhaustion markers LAYN (Ea), CTLA4 (Eb), and HAVCR2 (EC) in HCC patients.

In order to further understand the molecular characteristics of the infiltrated T cells in HCC patients, T cells were separated from the tumor and adjacent normal tissue samples obtained from the single‐cell RNA sequence (Figure 2A). It was observed that few T cells existed in the tumor tissues (Figure 2B). Subsequently, the monocle 2 R package was employed for the Pseudo‐Time analysis of exhausted T cells.^16^ Interestingly, in the process of T‐cell infiltration, the exhausted T cells that had infiltrated into the tumor tissues were those that had differentiated from the adjacent tissues (Figure 2C), and the T cell‐specific molecular markers of this sub‐population were completely reprogrammed during this infiltration process (Figure 2D). Importantly, the T‐cell exhaustion biomarkers (LAYN, CTLA4, and HAVCR2) were highly expressed in the tumor tissues (Figure 2E). Collectively, these data demonstrated that quantitatively and functionally exhausted T cells that had infiltrated into the tumor tissues were those that had differentiated from the adjacent tissues and then infiltrated into the tumor tissue in the HCC patients.

### 3.3 NGF‐NGFR communication inefficiency suppressed the mitotic spindle signaling pathway

The above‐stated results demonstrated that the NGF‐NGFR communication failure in HCC was driven by the differentiation process. However, the underlying mechanism of NGF‐NGFR communication in T‐cell exhaustion remains largely unclear. In order to better understand the molecular regulation mechanism and biological functioning of NGF and NGFR in tumor tissues, GSEA was performed using the pan‐cancer approach. The result revealed that overexpression of NGF and NGFR could activate apoptosis and the mitotic spindle pathway (Figures S1 and S2).

Furthermore, the TCGA‐LIHC RNA‐Seq data were divided into NGF/NGFR high‐expression and NGF/NGFR low‐expression groups of patients. Subsequently, 3576 differentially expressed genes (DEGs) were screened out successfully (Figure S3A) using the criteria |logFC| > 0.8 and *p* < 0.05 (Figure S3B). Next, the GO enrichment analysis was performed, which revealed immune receptor activity, growth factor activity, extracellular matrix organization, and extracellular structure organization, among others, as the main enriched processes (Figure S3C–E). Further, the GSEA assay results suggested that NGF‐NGFR communication was involved in the mitotic spindle signaling pathway (Figure S3F). In summary, these results indicated that NGF‐NGFR communication inefficiency might affect the immune response progression in HCC via the mitotic spindle signaling pathway.

### 3.4 NGF‐NGFR communication inefficiency induces mitotic arrest via the microtubule network

**FIGURE 3:**
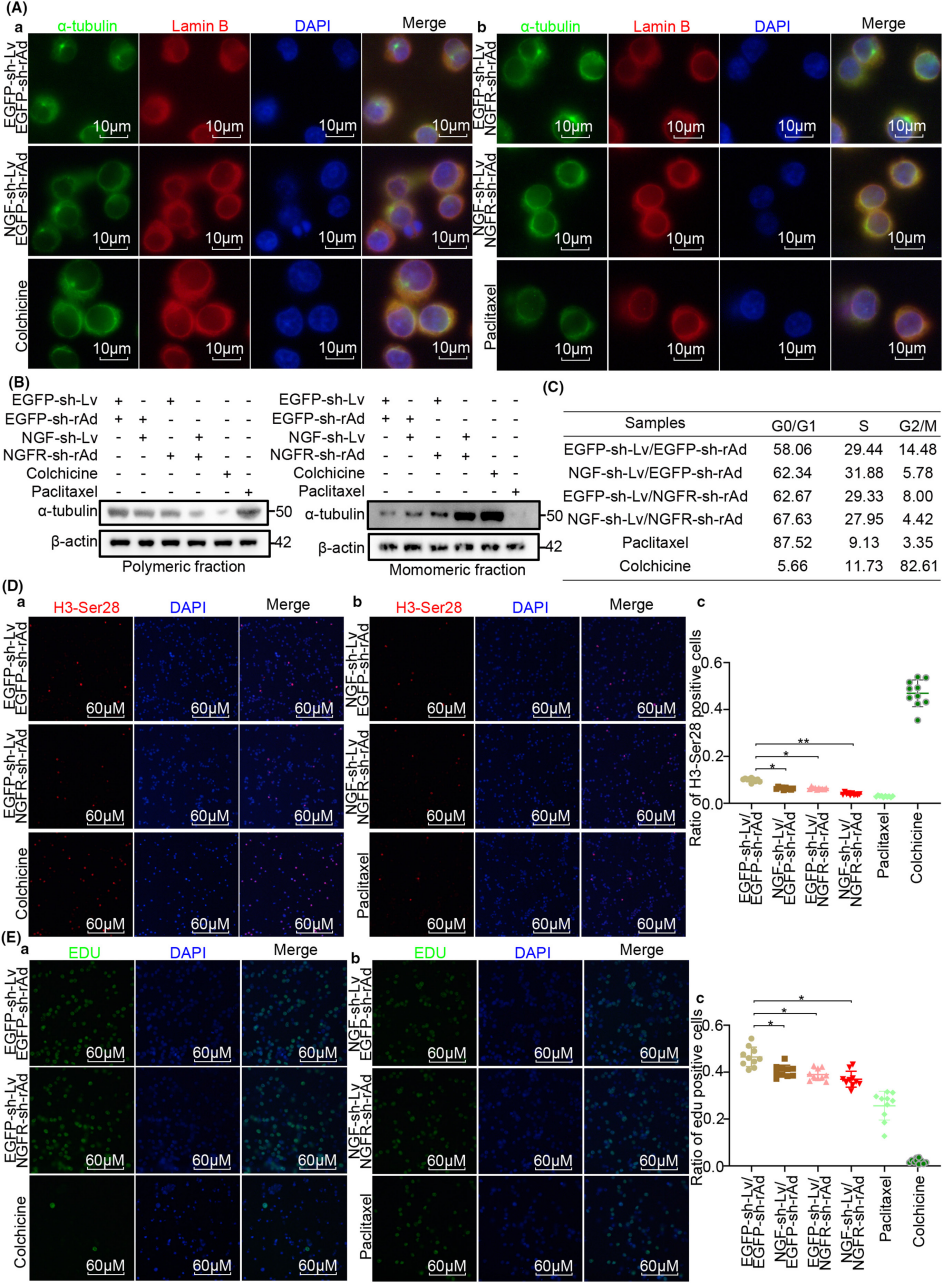
NGF‐NGFR communication inefficiency suppressed the organization of the mitotic spindle. (Aa, b) α‐tubulin (green) and Lamin B (red) staining for observing the organization of the mitotic spindle and the nuclear envelope in Jurkat T cells. Scale bar, 10 μm. (B) Immunoblot assay for examining the monomeric α‐tubulin and polymeric α‐tubulin in Jurkat T cells. (C) Flow cytometry assay for revealing the cell cycle distribution of Jurkat T cells. (Da, b) H3‐Ser28 (red) staining for determining the mitotic index for the Jurkat T cells. Scale bar, 60 μm. (Dc) Ratio of H3‐Ser28 positive cells. (Ea, b) Edu (green) staining for determining the cell proliferation of Jurkat T cells. Scale bar, 60 μm. (Ec) Ratio of Edu‐positive cells. Genomic DNA was stained in blue using the DAPI solution. *n* = 12, *p < 0.05, **p < 0.01.

The preliminary findings of the present study suggested that the mitotic spindle could be regulated by NGF‐NGFR communication. Therefore, NGF‐stable and low‐expression Huh7 cells and NGFR‐transient and low‐expression Jurkat T cells were infected with NGF‐shRNA‐Lv and NGFR‐shRNA‐rAd, respectively (Table S5). The microtubule network in the Jurkat T cells, collected from the Huh7 and Jurkat T co‐cultured system, was incubated with an anti‐α‐tubulin antibody for immunofluorescence staining. The microtubule network in the Jurkat T cells from the NGF and NGFR low expression co‐culture system presented a typical arrangement in most of the cells, while cells in the NGF and NGFR low expression co‐culture system or those in the group exposed to colchicine and paclitaxel exhibited an irregular bipolar array kind of arrangement. Consistently, in the NGF/NGFR low expression system or the cells exposed to colchicine and paclitaxel, a small nuclear envelope was observed following the anti‐lamin B staining (Figure 3A). Further, monomeric tubulin and polymeric tubulin were extracted to determine the organization of tubulin. As depicted in Figure 3B, the polymeric tubulin levels decreased following the NGF/NGFR interference or upon colchicine exposure, and this phenomenon was reversed upon paclitaxel exposure. These results suggested that NGF‐NGFR communication inefficiency inhibited mitotic spindle formation through the inhibition of microtubule aggregation.

It is widely accepted that mitotic spindle organization is necessary for the progression of the cell cycle.^17^ Therefore, the flow cytometry analysis of the cell cycle, the mitotic index (H3‐Ser28 staining) evaluation, and EDU staining were performed simultaneously to investigate the NGF‐NGFR communication failure in cell proliferation regulation. As visible in Figure 3C, a G0/G1 phase cell cycle arrest occurred in the NGF/NGFR low‐expression system (58%–67%). Consistently, decreased H3‐Ser28‐ and EDU‐positive cells were observed in the NGF/NGFR low‐expression system (Figure 3D,E). These results indicated that NGF‐NGFR communication inefficiency suppressed cell proliferation through the inhibition of mitotic spindle formation.

### 3.5 NGF‐NGFR communication inefficiency suppressed mitotic spindle formation through the HDAC1 unclear trans‐localization inhibited PREX1 expression

**FIGURE 4:**
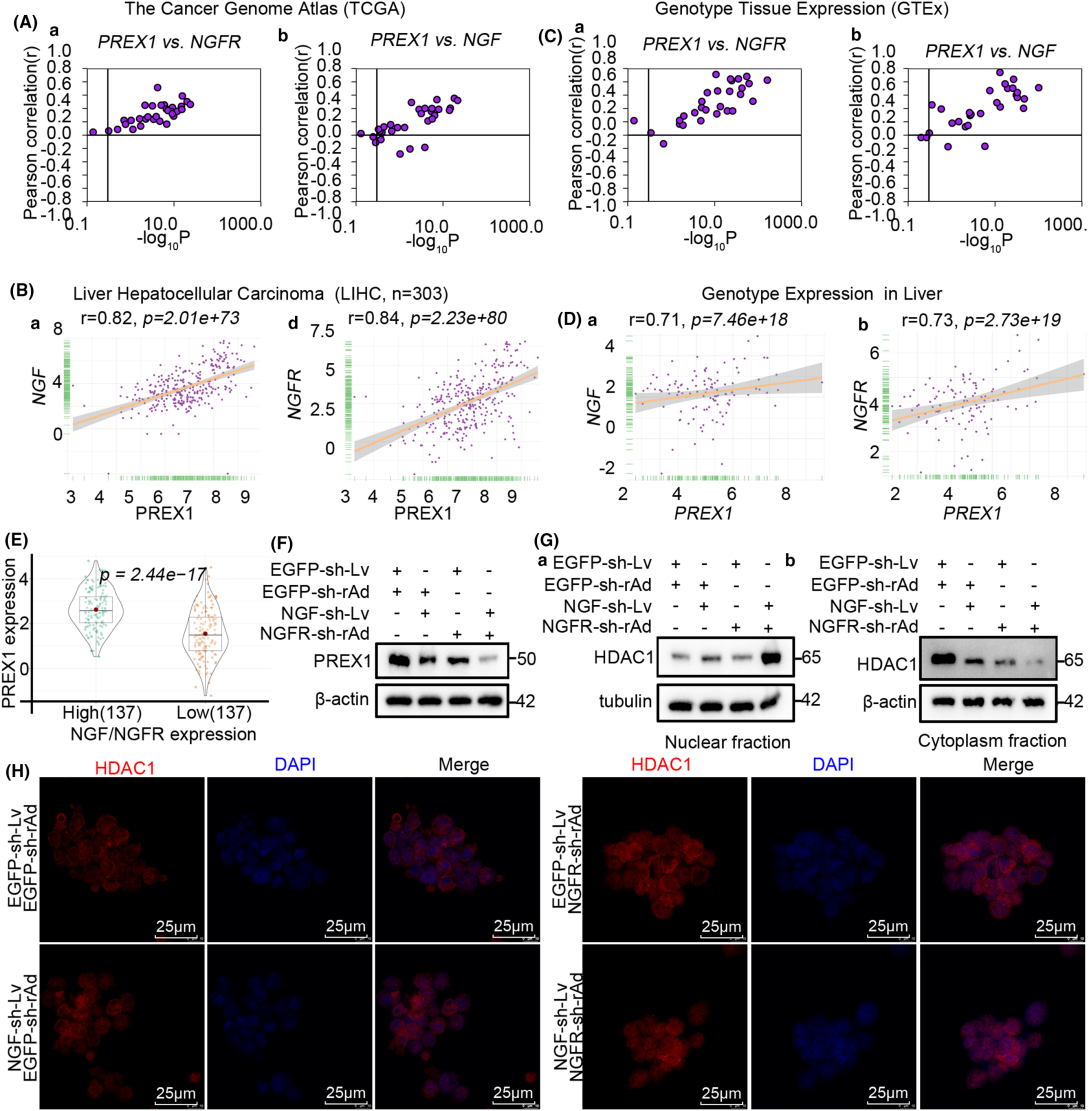
The molecular mechanism underlying the suppression of mitotic spindle formation is due to NGF‐NGFR communication inefficiency. (A) Co‐expression of PREX1 with NGF (Aa) and NGFR (Ab) in the tumors from TCGA. (B) Co‐expression of PREX1 with NGF (Ba) and NGFR (Bb) in the tumors from LIHC. (C) Co‐expression of PREX1 with NGF (Ca) and NGFR (Cb) in the tumors from GTEx. (D) Co‐expression of PREX1 with NGF (Da) and NGFR (Db) in the tumors from the liver. (E) Violin plot depicting the differences in the expression of PREX1 between NGF/NGFR low‐expression and high‐expression groups of tumor tissues. (F) The immunoblot assay for deciphering the effect of NGF‐NGFR communication inefficiency on PREX1 expression. (G) The immunoblot assay for determining the effect of NGF‐NGFR communication inefficiency on the nuclear translocation of HDAC1. Ga for the nuclear fraction and Gb for the cytoplasm fraction. (H) Immunofluorescence staining for determining the effect of NGF‐NGFR communication inefficiency on the nuclear translocation of HDAC1. Scale bar, 25 μm. Genomic DNA was stained with the DAPI solution.

Previous results of the present study suggested that NGF‐NGFR communication inefficiency suppressed T‐cell proliferation by affecting the organization of the mitotic spindle. However, the detailed molecular mechanism underlying this effect remains to be elucidated so far. Therefore, the gene expression of the mitotic spindle pathway regulated by the NGF‐NGFR communication (Table S5) was analyzed, and it was observed that NGF and NGFR were positively correlated with PREX1 in TCGA, LICH, GTEx, and the liver (Figure 4A–D). Consistently, PREX1 was downregulated in the NGF/NGFR low‐expression tumor tissues of HCC patients (Figure 4E) and also in the NGF and NGFR low‐expression co‐culture system (Figure 4F). However, PREX1 was suppressed through the deacetylation of histones at the promoter region with HDAC1.^18^ Therefore, the nucleus and cytoplasm were isolated from the cells of the NGF‐NGFR low‐expression co‐culture system using a commercial nucleus extraction kit. The results revealed that HDAC1 was non‐distinctly localized (Figure 4G), and a similar phenomenon was observed in the confocal immunofluorescence staining analysis of HDAC1 (Figure 4H). These results indicated that NGF‐NGFR communication inefficiency suppressed mitotic spindle formation through HDAC1 unclear trans‐localization‐inhibited PREX1 expression.

### 3.6 Overexpressed PREX1 reversed the inhibition of mitotic spindle organization due to NGF‐NGFR communication inefficiency

**FIGURE 5:**
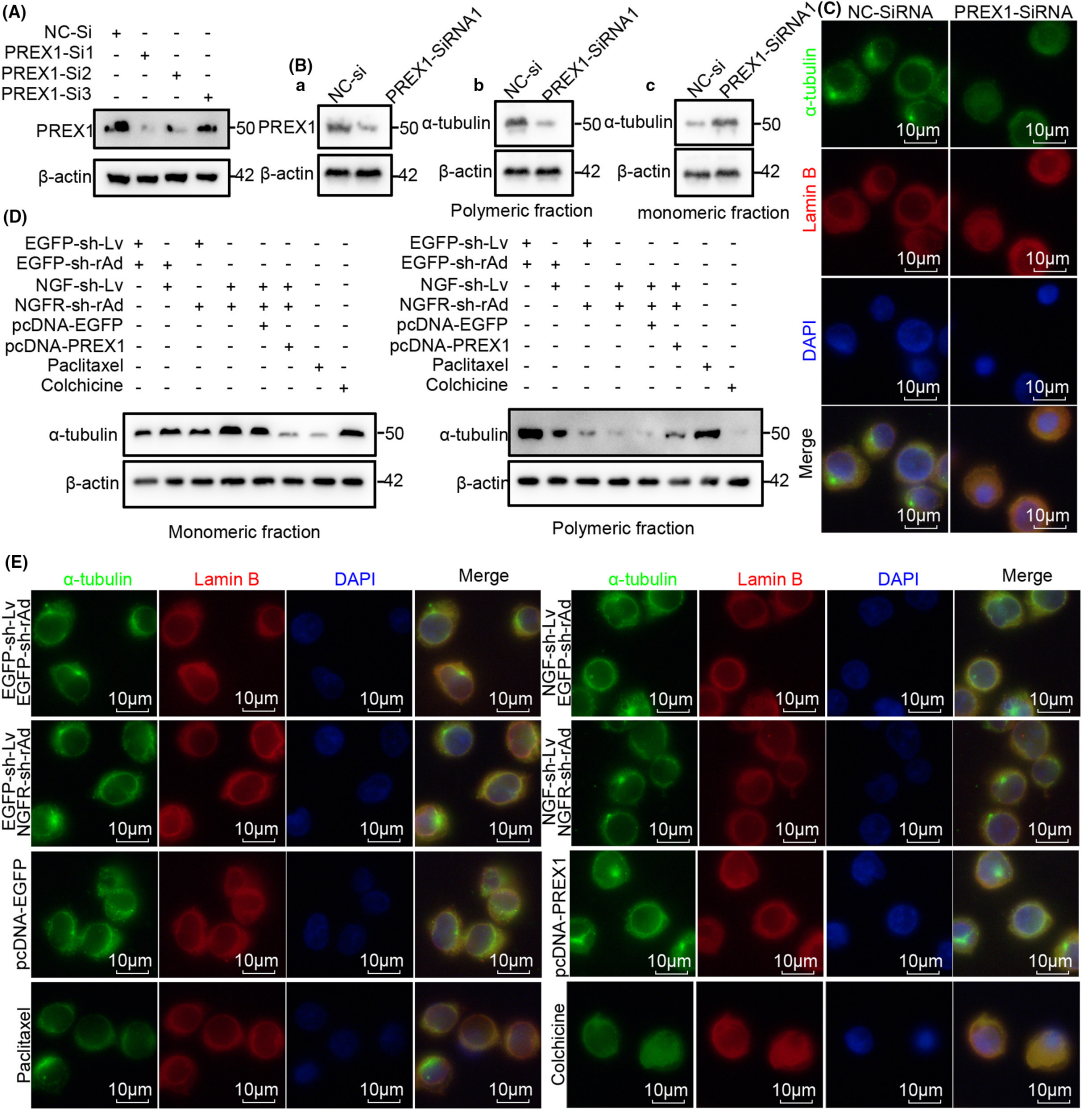
The role of PREX1 expression in NGF‐NGFR communication inefficiency‐suppressed mitotic spindle organization. (A) An immunoblot assay was performed to determine the effect of PREX1‐siRNA on PREX1 expression. As for the total fraction, Ab for the polymeric fraction, and Ac for the monomeric fraction. (B) Immunoblot assay for determining the impact of PREX1‐siRNA on the polymerization of α‐tubulin. (C) α‐tubulin (green) and Lamin B (red) staining results reveal the effect of PREX1‐siRNA on the organization of the mitotic spindle and the nuclear envelope. Scale bar, 10 μm. (D) An immunoblot assay was performed to determine the impact of PREX1 overexpression on NGF‐NGFR communication inefficiency‐suppressed polymerization of α‐tubulin. (E) α‐tubulin (green) and Lamin B (red) staining results reveal the effect of PREX1 overexpression on NGF‐NGFR communication inefficiency‐suppressed organization of the mitotic spindle. Scale bar, 10 μm. Genomic DNA was stained using the DAPI solution.

The above results indicated that NGF‐NGFR communication inefficiency inhibited the organization of the mitotic spindle and the PREX1 expression. However, this does not necessarily suggest that the inhibition of the mitotic spindle formation due to NGF‐NGFR communication inefficiency was induced by the suppression of PREX1. Therefore, three PREX1‐siRNAs were constructed and transfected into Jurkat T cells. The western blotting analysis was performed, and the results revealed that the PREX1 effectively interfered with PREX1‐siRNA1 (Figure 5A,Ba). Subsequently, monomeric tubulin and polymeric tubulin were extracted to determine the organization of tubulin. It was revealed that the interference of PREX1 expression effectively inhibited the organization of tubulin (Figure 5Bb,c), which was also confirmed in the immunofluorescence staining analysis (Figure 5C). Consistently, the results of EDU staining and the mitotic index (H3‐Ser28 staining) revealed that the interference of PREX1 expression suppressed cell proliferation (Figures S4A and S5A).

In order to further investigate the role of PREX1 expression inhibited organization of mitotic spindle in NGF‐NGFR communication inefficiency, the PREX1 expression in the NGF/NGFR low‐expression co‐culture system was reversed through the transfection of the pcDNA‐PREX1 plasmid. Western blotting and immunofluorescence staining were performed to determine the organization of α‐tubulin, and the results revealed that PREX1 expression effectively restored the polymerization of tubulin that was inhibited due to NGF‐NGFR communication inefficiency (Figure 5D,E). Similarly, cell proliferation suppressed due to NGF‐NGFR communication inefficiency was also restored upon PREX1 expression (Figures S4B and S5B). These results indicated that the NGF‐NGFR communication inefficiency inhibited the organization of tubulin through the inhibition of PREX1 expression and, thus, induced the inhibition of cell proliferation.

### 3.7 NGF‐NGFR communication inefficiency impaired the anti‐tumor immunotherapy with PD‐1 mAb in both mouse models and patients

**FIGURE 6:**
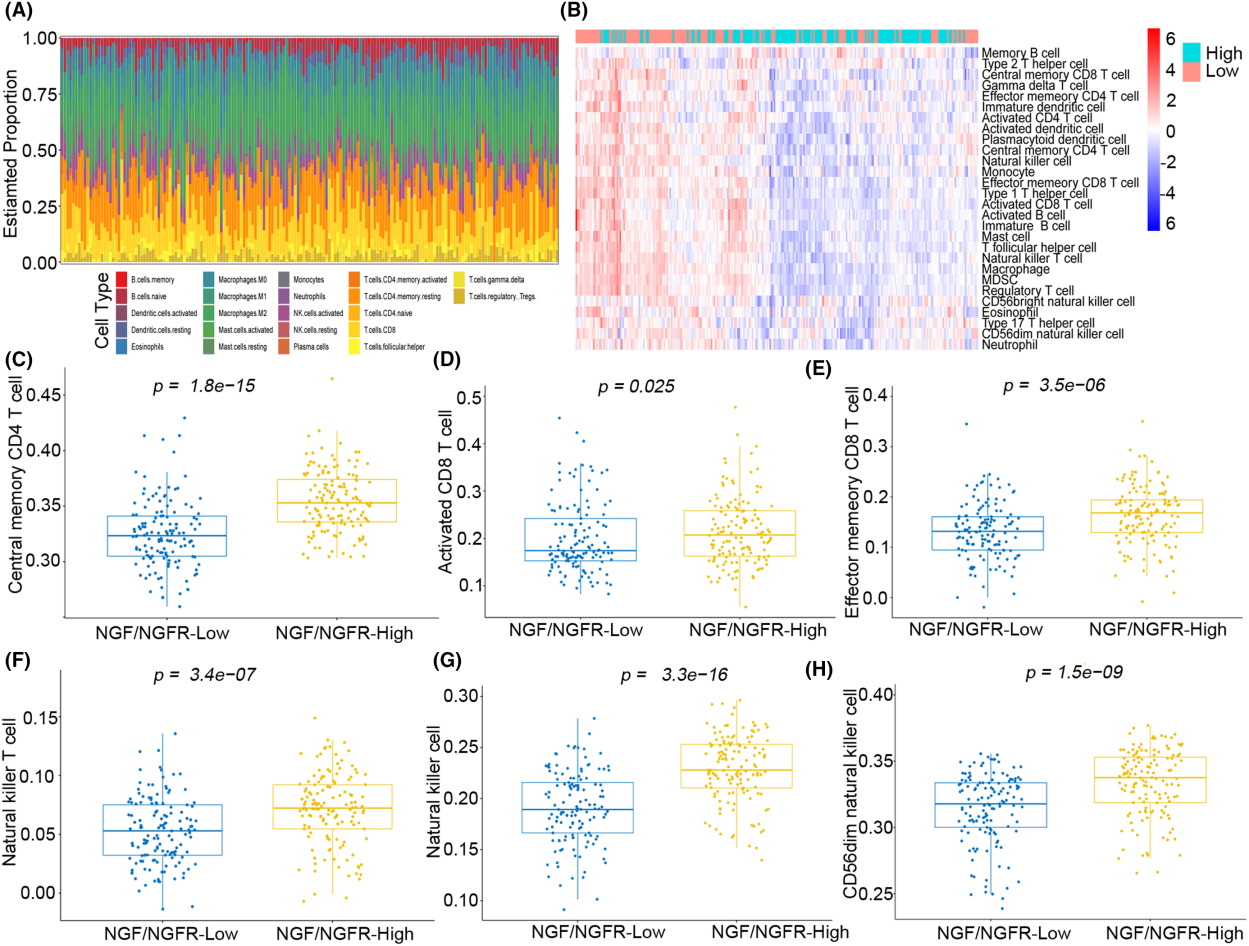
Landscape of immune infiltration in the NGF and NGFR high‐expression and low‐expression LIHC patients. (A) Bar plot illustrating immune infiltration in the NGF and NGFR high‐expression and low‐expression LIHC patients (B) Heatmap illustrating immune infiltration in the NGF and NGFR high‐expression and low‐expression LIHC patients. The proportions of (C) central memory CD4^+^ T cells, (D) activated CD8^+^ T cells, (E) effector memory CD8^+^ T cells, (F) natural killer T cells, (G) natural killer cells, (H) and CD56dim natural killer cells, infiltrated in the NGF and NGFR high‐expression and low‐expression LIHC patients.

The NGF‐NGFR communication inefficiency was demonstrated to suppress T‐cell proliferation in the tumors of HCC patients. However, which particular kind of immune cells is suppressed due to NGF‐NGFR communication inefficiency was unclear. The landscape of immune cells revealed that the memory CD4^+^ T cells, CD8^+^ T cells, and activated NK cells had infiltrated into the NGF expression LIHC patients (Figure S6), while only memory CD4^+^ T cells had infiltrated into the NGFR expression LIHC patients (Figure S7). Furthermore, immune cells that had infiltrated the tumor tissues, including the CD56dim natural killer cells, central memory CD4^+^ T cells, natural killer T cells, activated CD8^+^ T cells, natural killer cells, and effector memory CD8^+^ T cells, were downregulated in the NGF/NGFR low‐expression group of patients (Figure 6). These results indicated that NGF‐NGFR communication inefficiency inhibited the immune cell infiltration in the tumor tissues of patients.

**FIGURE 7:**
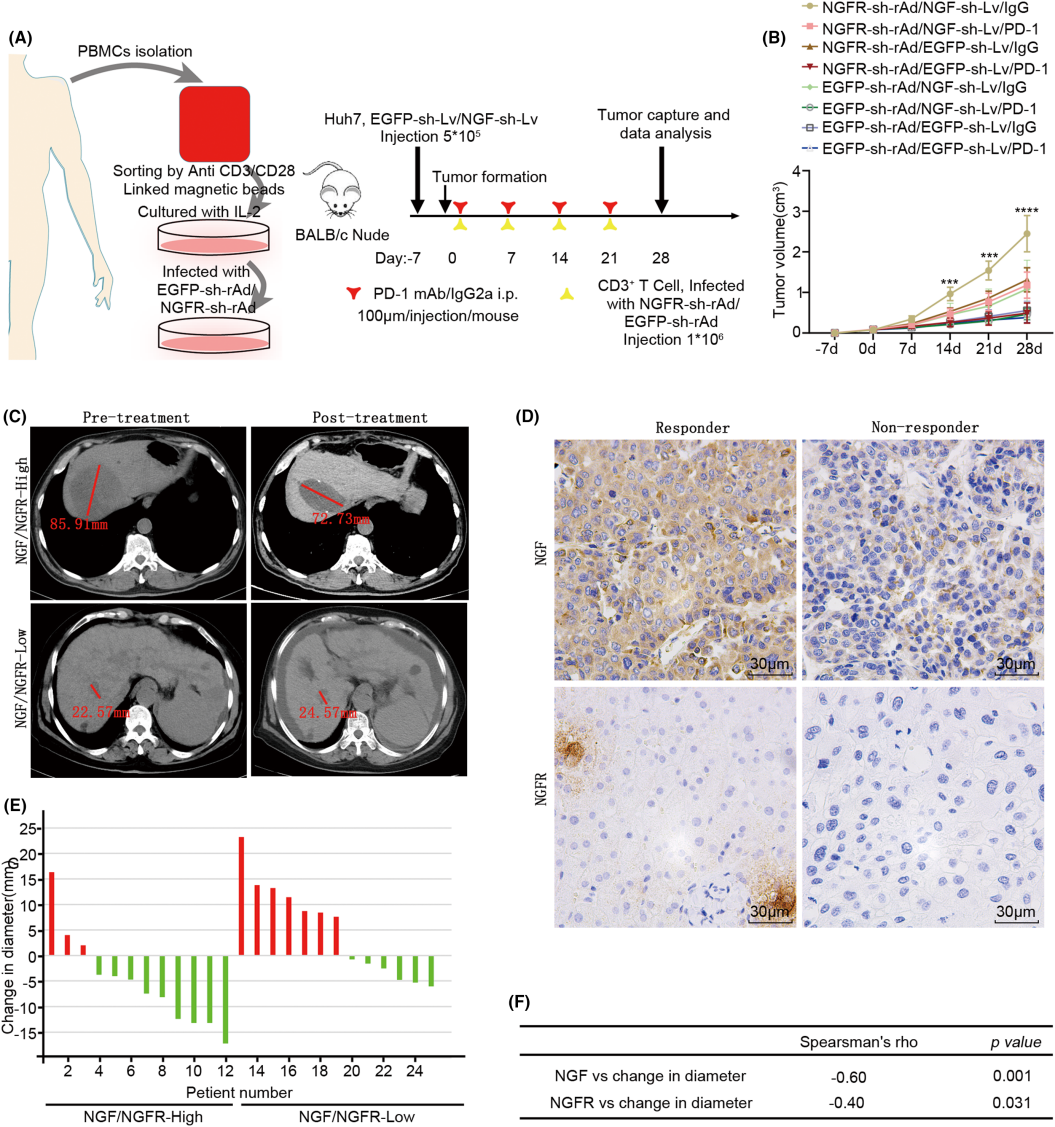
Synergistic role of NGF‐NGFR communication and PD‐1 mAb. (A, B) Immunodeficient mice, rebuilt with NGFR low‐expression CD3^+^ T cells and exposed to PD‐1 mAb therapy, were injected with NGFR‐shRNA‐Lv‐infected Huh7 cells. (A) The outline of the therapeutic plan for the immunodeficient mice. (B) Tumor growth curves are measured once a week. (C) CT imaging depicting the tumor diameter (red line). (D) NGF and NGFR expressions were recorded using the immunohistochemistry staining of the tumor tissues of HCC patients. Scale bar, 30 μm. (E) Tumor diameter in HCC patients before and after the PD‐1 mAb therapy. (F) Spearman's rank correlation for determining the diameter change and the NGF and NGFR expressions.

In patients, the PD‐1/PDL‐1 interaction was demonstrated to induce the functional exhaustion of T cells by inhibiting cell proliferation and activation,^19^ while the NGF‐NGFR communication was observed to be positively correlated with the expressions of PD‐L1 and PD‐1 in TCGA, LIHC, and GTEx (Figure S8). Therefore, to understand the role of NGF‐NGFR communication in the anti‐tumor immunotherapy with PD‐1 mAb, the BALB/c nude mice were injected subcutaneously with the NGF‐sh‐Lv‐infected Huh7 cells. After the successful formation of the subcutaneous tumor, the immune system was reconstructed via an injection of CD3^+^ T cells (1*10^6^), which had been separated from PBMCs and transfected with NGFR‐sh‐rAd, and PD‐1 mAb (Figure 7A). Decreased levels of CD3^+^ cells and CD8^+^ cells (Figure S9) and increased tumor growth (Figure 7B) were observed in the IgG mAb‐exposed and NGF‐sh‐Lv/NGF‐sh‐rAd‐injected mice. These results suggested that NGF‐NGFR communication inefficiency impaired the PD‐1 mAb therapy for tumor inhibition in the mouse model.

In order to further understand the role of NGF‐NGFR communication in PD‐1 mAb anti‐tumor immunotherapy in HCC patients (Table S2), NGF‐NGFR communication was studied in 29 PD‐1‐positive patients exposed to PD‐1 mAb, 12 non‐responders, and 17 responders. Two typical cases of NGF and NGFR expression and change in the tumor diameter (red line) upon PD‐1 mAb anti‐tumor immunotherapy are presented in Figure 7C,D. Importantly, the NGF and NGFR low‐expression patients were from the PD‐1 mAb non‐responders (Figure 7E), and the changed diameter was positively correlated with NGF and NGFR expressions (Figure 7F). These results suggested that the tumor inhibition ability of PD‐1 mAb anti‐tumor immunotherapy was weakened due to the NGF‐NGFR communication inefficiency in the tumor tissues of HCC patients.

### 3.8 NGF‐NGFR communication inefficiency resulted in incursive clinicopathological characteristics and disappointing prognosis in HCC patients

**FIGURE 8:**
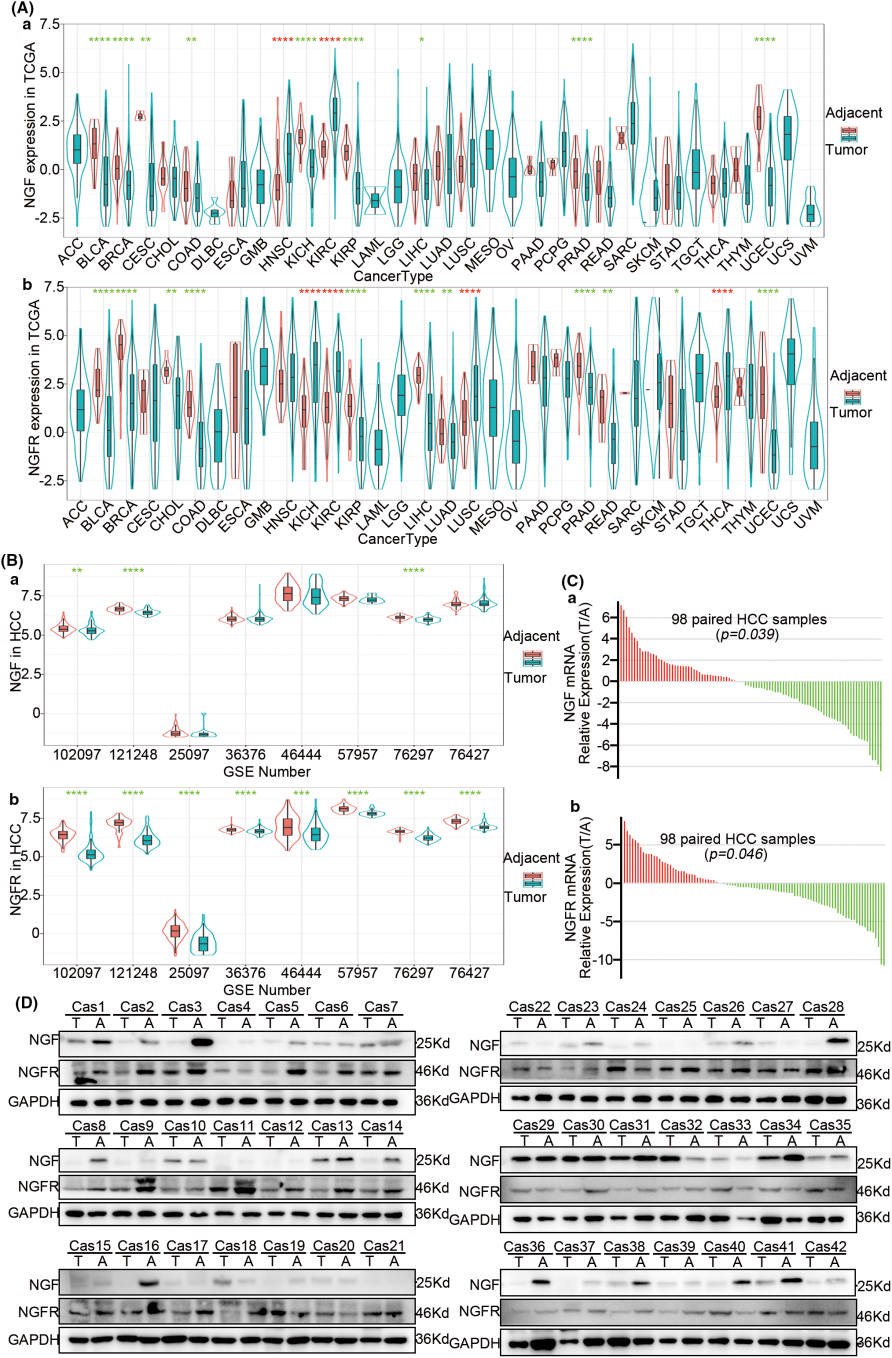
NGF‐NGFR communication in HCC patients. (Aa) NGF and (Ab)NGFR expressions in the patient data from TCGA. (Ba) NGF and (Bb) NGFR expressions in HCC patient data from GEO. (C) The relative mRNA expressions of NGF (Ca) and NGFR (Cb) in the data of 98 HCC patients were expressed as −ΔΔCT values. (D) The relative protein contents of NGF and NGFR in paired tumors and adjacent tissues of 42 HCC patients were determined using western blotting.

To unravel further details of the NGF‐NGFR communication in HCC patients, 33 cancer types and nine HCC RNA‐seq datasets were collected and evaluated for NGF and NGFR expressions. A downregulation of NGF was observed in the tumor tissues of BLCA, BRCA, CESC, COAD, KICH, KIRP, LIHC, PRAD, and UCEC, while NGFR was downregulated in BLCA, BRCA, CHOL, COAD, KIRP, LIHC, LUAD, PRAD, READ, STAD, and UCEC (Figure 8A). In the HCC RNA‐seq datasets, NGF was significantly downregulated in three RNA‐seq datasets, while NGFR expression was low in all RNA‐seq datasets (Figure 8B). Consistently, the downregulation of NGF and NGFR in the tumor tissue of HCC patients was observed in the qRT‐PCR analysis (Table S3; Figure 8C) and western blotting (Figure 8D) as well. These results suggested an impaired NGF‐NGFR communication in the tumor tissues of these patients.

To further elucidate the contribution of NGF‐NGFR communication inefficiency, the correlation of the downregulated NGF and NGFR with the survival of HCC patients was evaluated. The low expression NGF/NGFR group patients presented poor overall survival (31 vs. 22 months, *p* = 0.023), reduced time to disease‐free survival (31 vs. 22 months, *p* = 0.033), reduced disease‐free interval (24 vs. 17 months, *p* = 0.01), and worse progression‐free survival (21 vs. 15 months, *p* = 0.0048) (Figure S10A–D). A similar result was obtained for the HCC patients in the low expression NGF or NGFR patients (Figure S5). In addition, the multivariate analysis revealed NGF and NGFR levels, BMI scores, virus infection, and tumor stage as the independent risk factors for OS, DSS, DFI, and PFI (Figure S10E–H). Collectively, these results suggested that the frequently low‐expressed NGF and NGFR were correlated with incursive clinicopathological characteristics and disappointing prognosis in HCC patients.

## 4 DISCUSSION

In this context, the present study demonstrated that: (i) the T‐cell infiltration process from adjacent tissues to tumor tissue induces the failure of NGF‐NGFR communication; (ii) the NGF‐NGFR communication inefficiency suppresses the mitotic spindle formation during cell proliferation through HDAC1 unclear trans‐localization‐inhibited PREX1 expression; (iii) the NGF‐NGFR communication inefficiency impairs the PD‐1 mAb anti‐tumor immunotherapy in both mouse model and HCC patients; (iv) the expressions of both NGF and NGFR were low in the tumor tissues and were predictive of incursive clinicopathological features and disappointing prognosis. These results of the present study implicated that NGF‐NGFR communication inefficiency induces quantitative exhaustion of the T cells infiltrated in the tumor tissues, which limits the therapeutic effects of PD‐1 mAb. These findings present a novel direction for the research on anti‐tumor immunotherapy for the treatment of HCC.

**FIGURE 9:**
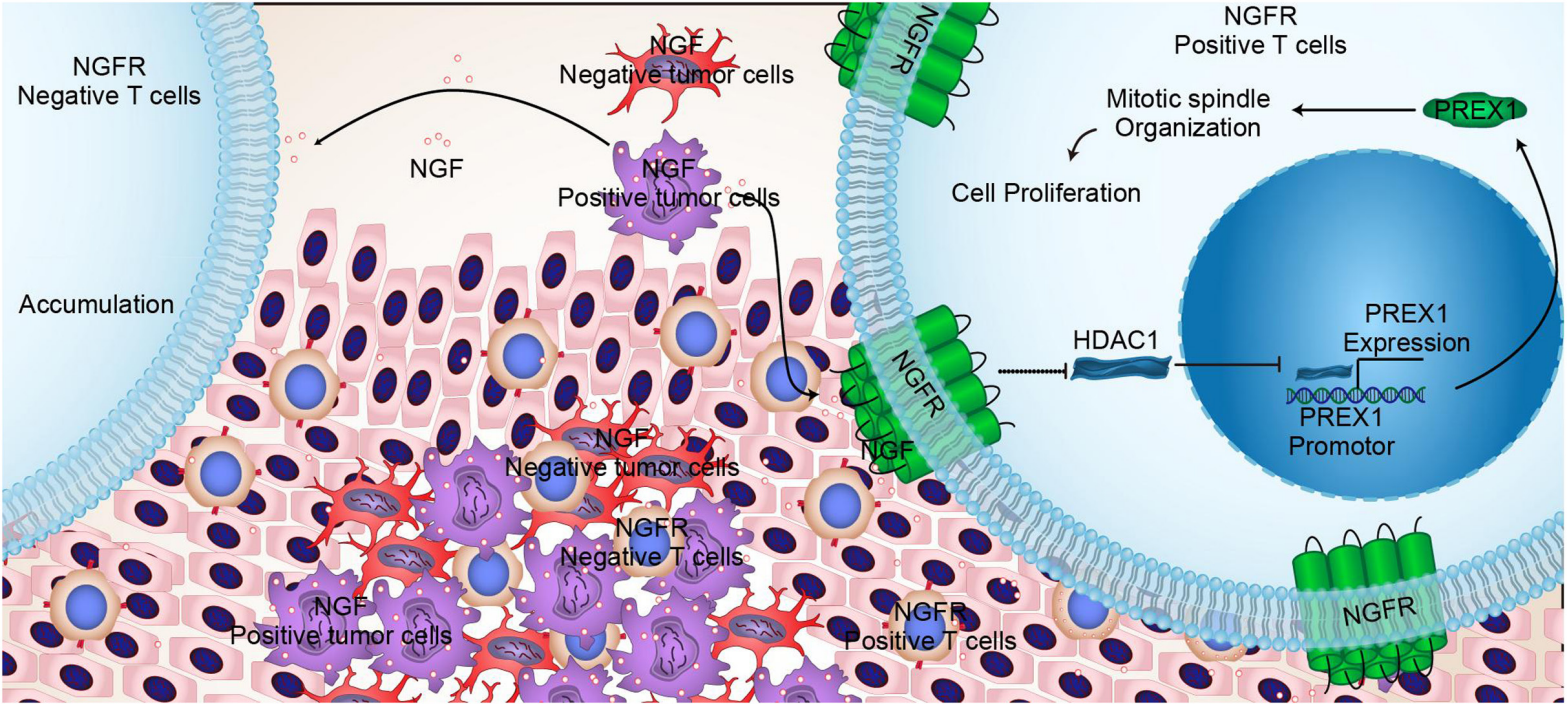
The molecular mechanism underlying the NGF‐NGFR communication inefficiency suppressed mitotic spindle signaling pathway activation in T‐cell proliferation.

Exhausted T cells infiltrated in the tumor tissue exhibit a reprogrammed expression profile for the continuous stimulation of the tumor antigen. Various studies have suggested that the markers of T‐cell exhaustion, such as CTLA‐4, BTLA, LAG‐3, 2B4, TIM‐3, CD160, and PD‐1, induce the functionally impaired state and thereby suppress the proliferation of the exhausted T cells in tumor tissues.^20^, ^21^ However, the relevant functional role and the molecular mechanism of cell proliferation on the quantitative exhaustion of infiltrated T cells are poorly understood so far. In this context, the present study revealed that NGF^+^ tumor cells, NGF^−^ tumor cells, NGFR^+^ T cells, and NGFR^−^ T cells appeared simultaneously in HCC patients. The NGF secreted from the tumor cells communicated with the NGFR present on the membranes of T cells, which promoted T‐cell proliferation through the activation of the mitotic spindle signaling pathway (Figure 9). Unfortunately, the expressions of NGF and NGFR were low in the tumor tissues of HCC patients, which could be the possible reason for the reduced numbers of T cells in these tissues. Moreover, NGF and NGFR were identified as low‐risk factors for the survival of HCC patients, and the low expression of NGF and NGFR was predictive of a poor prognosis (Figure S11).

NGFR present on the cell surface reportedly recognizes the NGF, BDNF, NT‐3, and NT‐4 signals that regulate various biological functions of the cells.^22^, ^23^ The cell‐to‐cell communication assay conducted in the present study revealed that NGF‐NGFR communication was the only signal in the tumor tissues of HCC patients. This result indicated no possibility of communication between NGFR and the other three ligands. Importantly, in the NGF‐NGFR communication, NGF was secreted from tumor cells while NGFR was expressed in the T cells. The advancements in the single‐cell sequencing technology were used in the present study, and the results revealed a unique NGF‐NGFR communication and precisely elucidated the cell types involved in the NGF‐NGFR communication in the tumor tissues of HCC patients. Accordingly, it is possible to precisely locate the tumors and T cells with impaired NGF‐NGFR communication, and this could serve as a novel molecular mechanism based on which the quantitative exhaustion of T cells could be elucidated. Therefore, the present study presents the possibility of effectively improving the quantitative exhaustion of T cells infiltrated into the tumor tissues.

It is reported that the regulation of biological functions via NGF‐NGFR communication depends on the status of TrkA in cells.^24^, ^25^, ^26^ In the present study, it was demonstrated that NGF‐NGFR communication inefficiency suppressed the proliferation of exhausted T cells infiltrated in the tumor tissues through the inhibition of mitotic spindle organization, although the role of TrkA status in the inhibition of cell proliferation via NGF‐NGFR communication inefficiency was not elucidated. Rather, a novel molecular mechanism underlying the role of NGF‐NGFR communication in the regulation of biological functions was revealed, according to which NGF‐NGFR communication inefficiency suppressed the organization of the mitotic spindle through HDAC1 unclear trans‐localization‐inhibited PREX1 expression, which ultimately promoted the quantitative exhaustion of T cells infiltrated in tumor tissues. This finding would provide a new research on the regulation of the proliferation of T cells infiltrated into the tumor tissues of HCC patients.

It is reported that the exhaustive molecular marker, PD‐1, which is expressed on the surface of exhausted T cells, impaired the tumor‐killing effects in tumor tissues.^27^, ^28^ Therefore, the immune checkpoint blocker PD‐1 mAb is the most widely used reagent for anti‐tumor immunotherapy in clinical practice.^29^, ^30^, ^31^ However, before performing the PD‐1 mAb therapy, positive detection of PD‐1 is necessary.^32^, ^33^ Moreover, numerous studies have suggested that the number of T cells in the tumor tissue directly affects the outcome of the PD‐1 mAb anti‐tumor immunotherapy,^34^, ^35^ implicating that the existence of T cells is essential for anti‐tumor immunotherapy. In the present study, NGF/NGFR was correlated positively with PD‐1/PDL‐1 expression in both tumor tissue and healthy tissue, and the NGF‐NGFR communication inefficiency was demonstrated to suppress the proliferation of T cells. These results implicated that NGF‐NGFR communication could simultaneously induce the proliferation of T cells and promote the proliferation of exhausted T cells. Interestingly, NGF‐NGFR communication and PD‐1 mAb acted synergistically to provide anti‐tumor immunotherapy. Therefore, NGF‐NGFR communication and the immune checkpoint blocker PD‐1 mAb could exhibit a synergistic effect in the tumor suppression procedure.

The present study elucidated NGF‐NGFR communication inefficiency in the tumor tissues of HCC patients. NGF‐NGFR communication inefficiency suppressed mitotic spindle formation via HDAC1 unclear trans‐localization‐inhibited PREX1 expression, thereby suppressing the proliferation of T cells that had infiltrated into the tumor tissues of HCC patients. NGF‐NGFR communication was correlated positively with PD‐1/PDL‐1 and exhibited synergistic action with PD‐1 mAb‐suppressed tumor progression in both mouse models and patients. This novel molecular mechanism underlying the NGF‐NGFR communication in T‐cell proliferation could provide fresh insights for anti‐tumor immunotherapy.

## AUTHOR CONTRIBUTIONS

**Xin Wang:** Data curation (equal); methodology (equal). **Tongwang Yang:** Formal analysis (equal); investigation (equal); methodology (equal); writing – original draft (equal). **Shangheng Shi:** Conceptualization (equal); data curation (equal); formal analysis (equal). **Chuanshen Xu:** Investigation (equal); methodology (equal). **Feng Wang:** Data curation (equal); formal analysis (equal); investigation (equal). **Deshu Dai:** Data curation (equal); formal analysis (equal); methodology (equal). **Ge Guan:** Data curation (equal); software (equal); validation (equal). **Yong Zhang:** Resources (equal); software (equal); supervision (equal). **Shuxian Wang:** Software (equal); validation (equal). **Jianhong Wang:** Data curation (equal); software (equal). **Bingliang Zhang:** Software (equal). **Peng Liu:** Validation (equal). **Xiaoshuai Bai:** Investigation (equal). **Yan Jin:** Investigation (equal). **Xinqiang Li:** Formal analysis (equal). **Cunle Zhu:** Investigation (equal). **dexi chen:** Resources (equal); supervision (equal). **Qingguo Xu:** Project administration (lead); resources (equal); supervision (equal); writing – review and editing (equal). **Yuan Guo:** Funding acquisition (equal); investigation (equal); methodology (equal); project administration (equal); supervision (equal); writing – review and editing (equal).

## FUNDING INFORMATION

The National Natural Science Foundation of China (82272973, 81900575); the Beijing Municipal Natural Science Foundation and Beijing Municipal Education Commission (KZ202010025037); the China Postdoctoral Science Foundation (2020M672003, 2022T150341); the Youth Science Foundation of Affiliated Hospital of Qingdao University (QYFY‐2021‐36) supported the current artwork.

## CONFLICT OF INTEREST STATEMENT

The authors declare that they have no conflict of interest.

## ETHICS STATEMENT

The ethics committee of the Affiliated Hospital of Qingdao University approved the project (QYFYWZLL27133). All individual participants or families were thoroughly informed regarding the study and were asked to sign a written informed consent form. Samples were obtained with informed consent from patients or family members. The guidelines of the National Institutes of Health and the Qingdao University Animal Care Facility were followed in the animal project.

## CONSENT FOR PUBLICATION

All authors provide their consent for publication of this article.

## Supporting information


Figure S1.

Figure S2.

Figure S3.

Figure S4.

Figure S5.

Figure S6.

Figure S7.

Figure S8.

Figure S9.

Figure S10.

Figure S11.

Table S1.

Table S2.

Table S3.

Table S4.

Table S5.

Data S1.

## Data Availability

The datasets used or analyzed during the current study are available from the corresponding author upon reasonable request.
