# Supplementary figures and images for: Heterogeneity‐induced NGF‐NGFR communication inefficiency promotes mitotic spindle disorganization in exhausted T cells through PREX1 suppression to impair the anti‐tumor immunotherapy with PD‐1 mAb in hepatocellular carcinoma
Source: Cancer Med. 2024 Jan 10;13(3):e6736. doi: 10.1002/cam4.6736 (PMC10905245; doi:10.1002/cam4.6736)

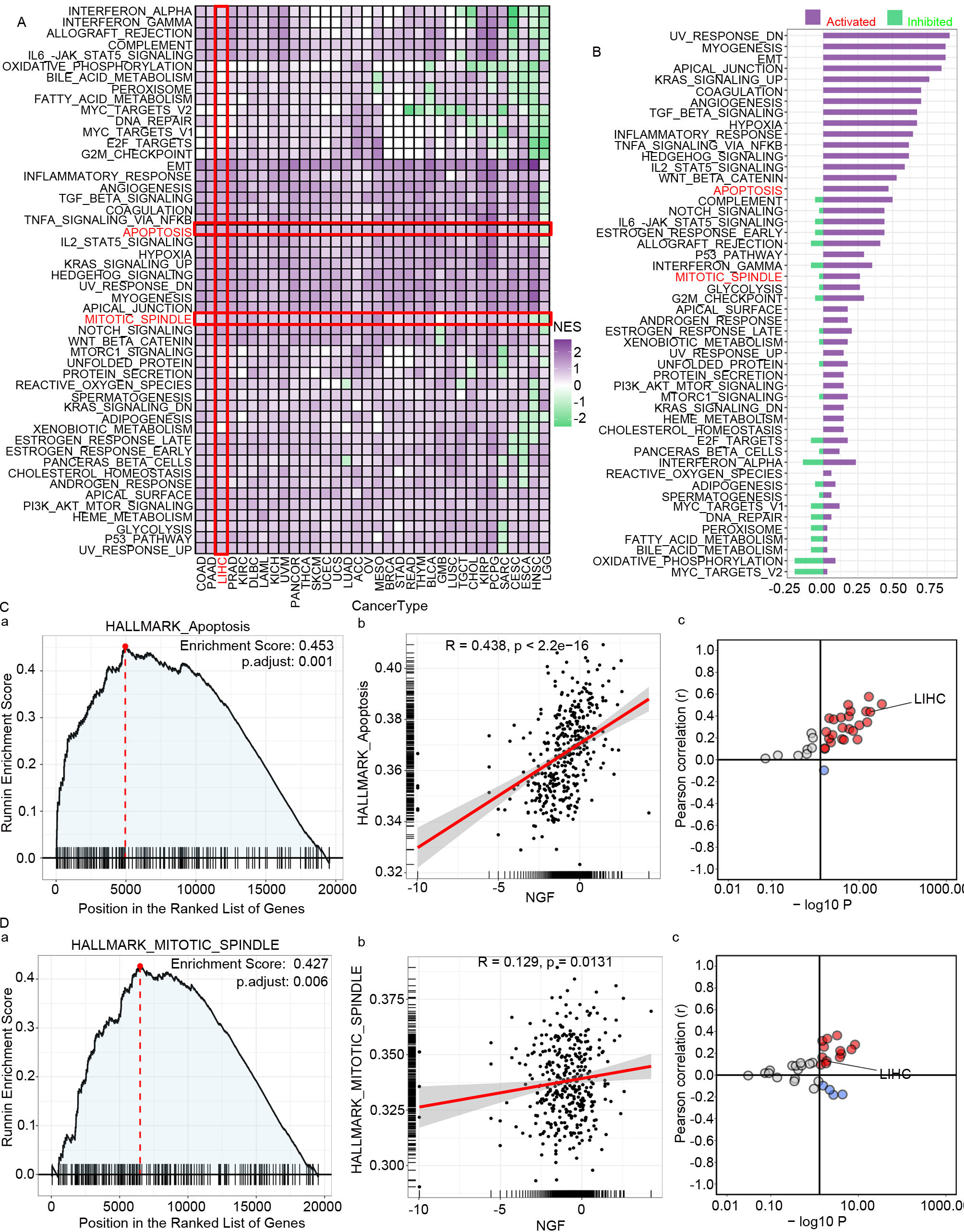

Supplement: Supplementary file 1 — Figure S1. [file CAM4-13-e6736-s013.tif]

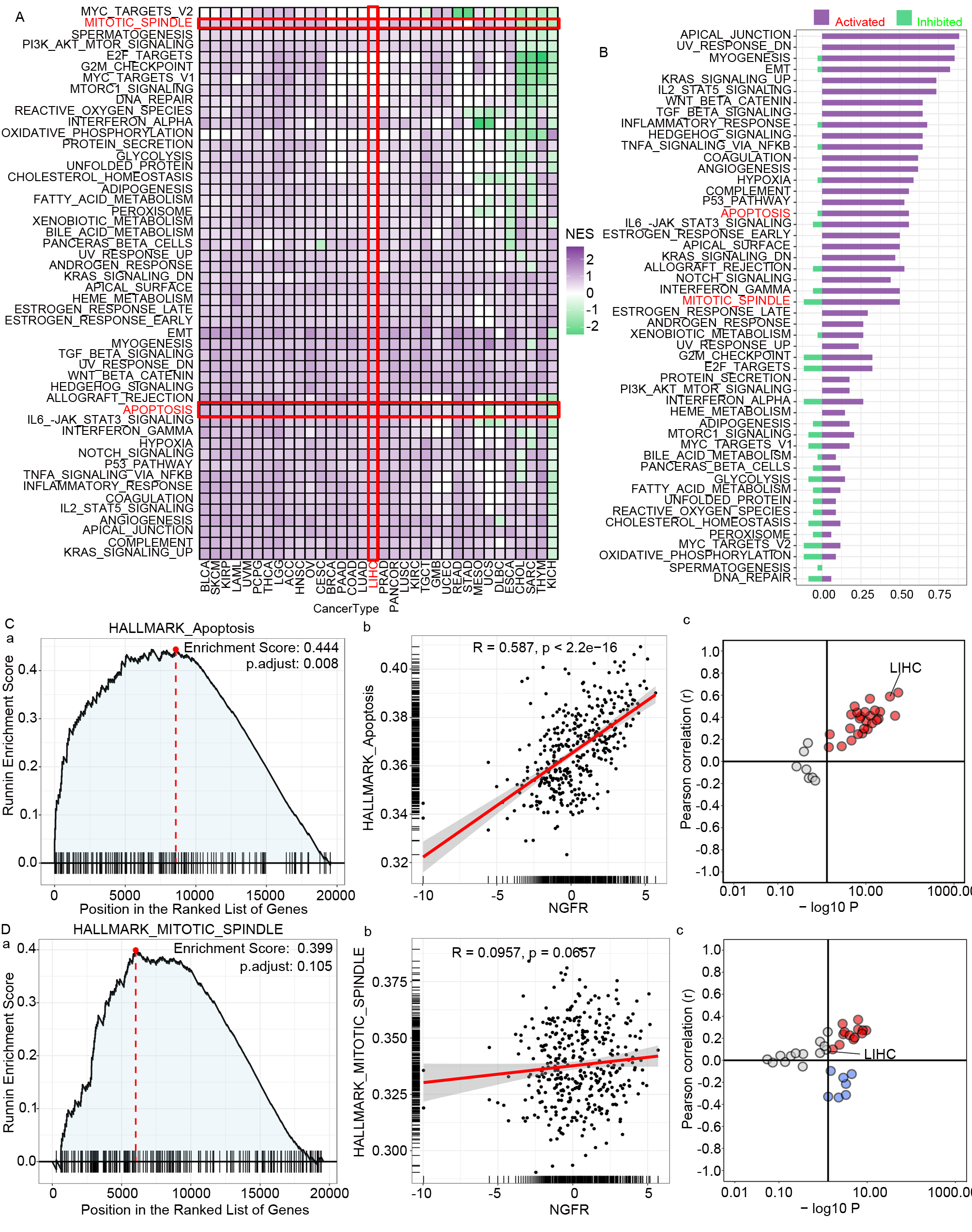

Supplement: Supplementary file 2 — Figure S2. [file CAM4-13-e6736-s011.tif]

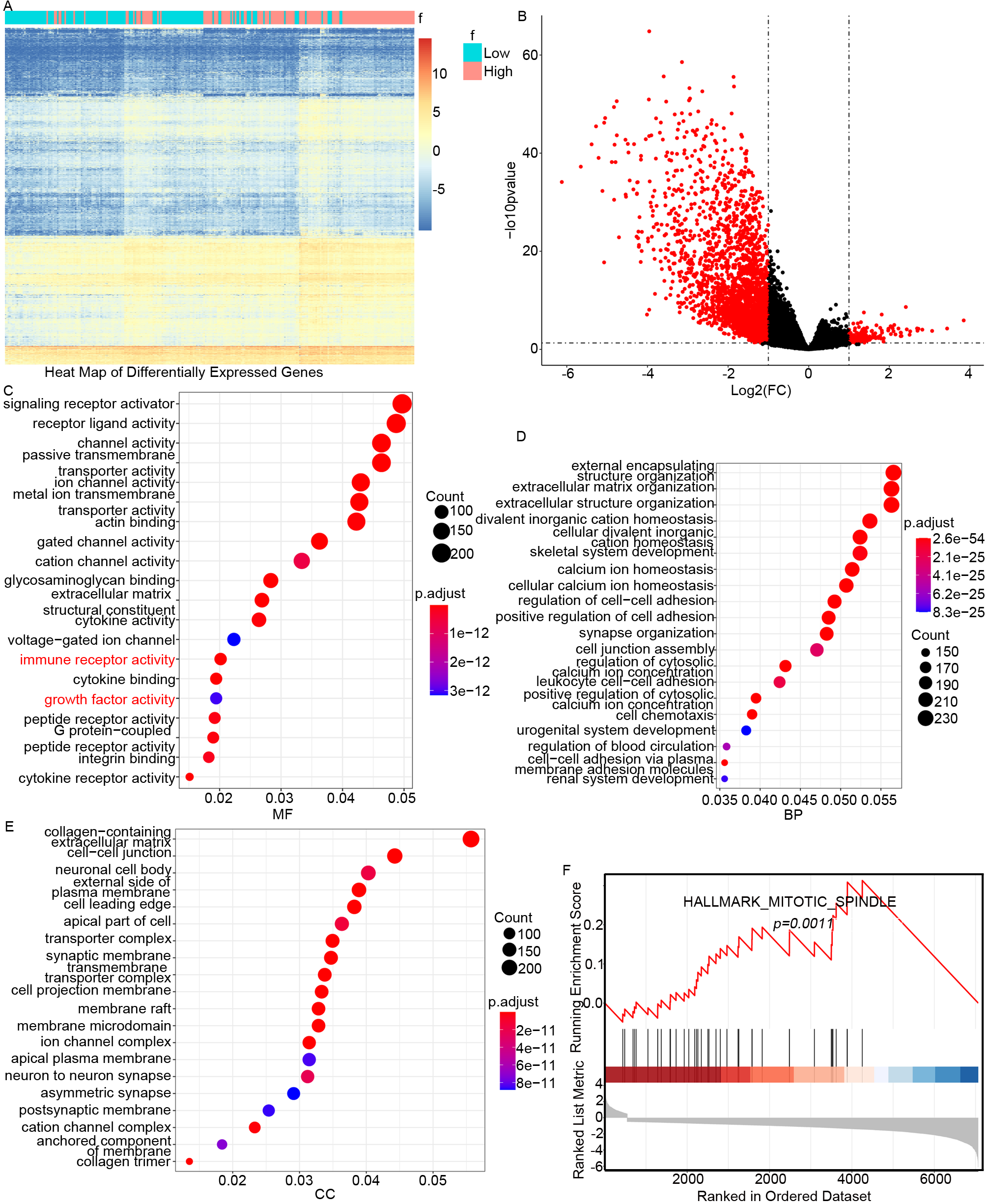

Supplement: Supplementary file 3 — Figure S3. [file CAM4-13-e6736-s002.tif]

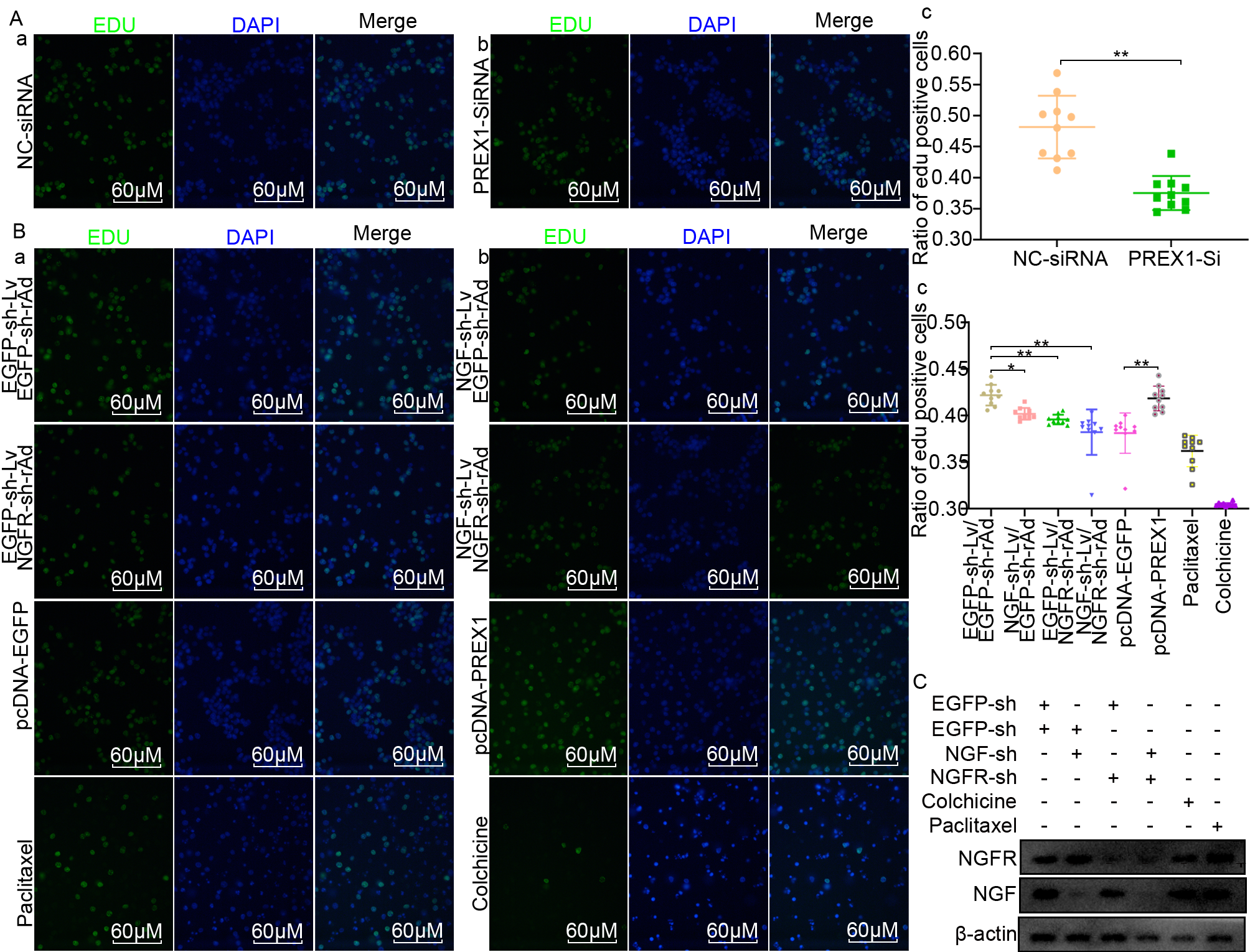

Supplement: Supplementary file 4 — Figure S4. [file CAM4-13-e6736-s014.tif]

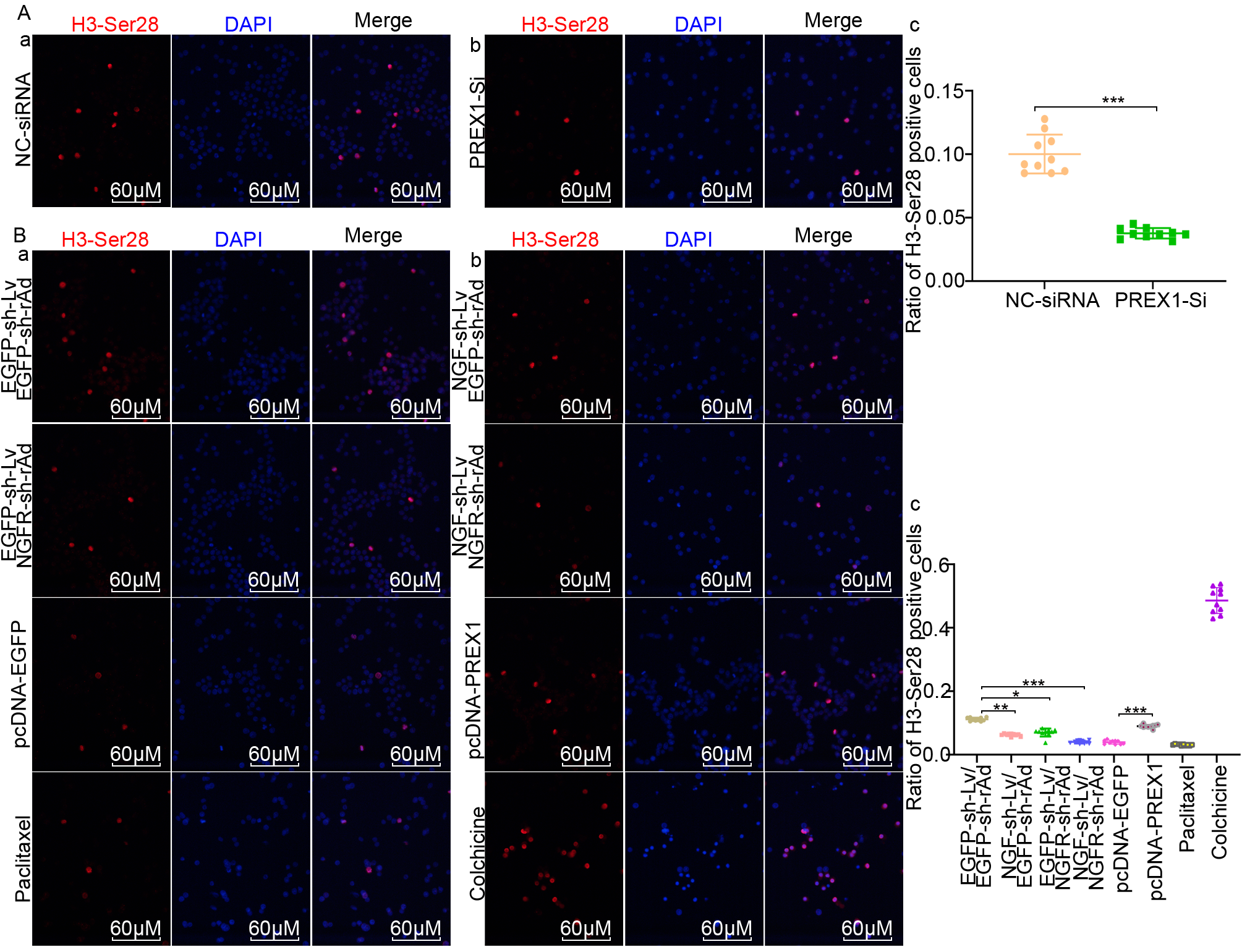

Supplement: Supplementary file 5 — Figure S5. [file CAM4-13-e6736-s017.tif]

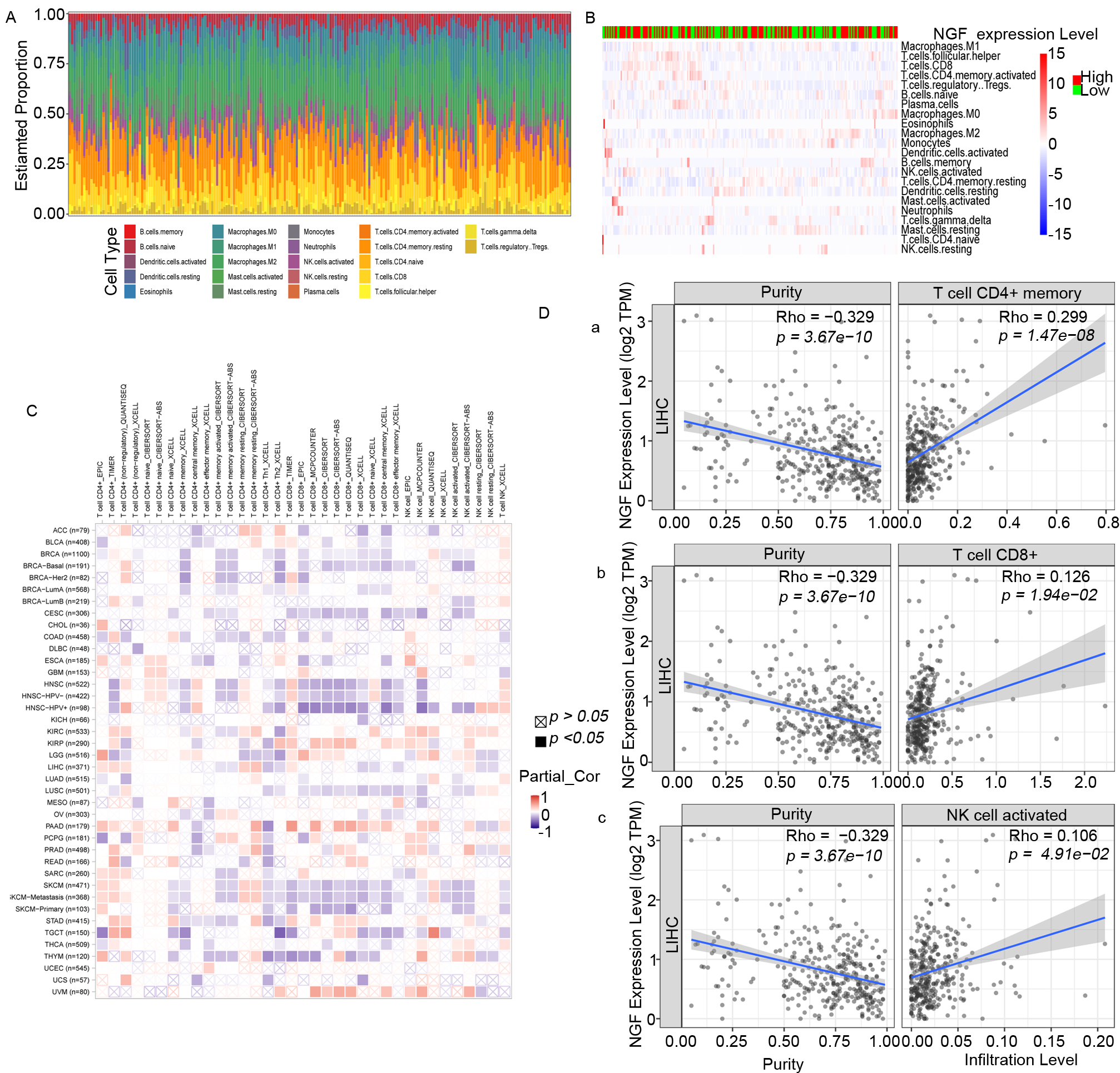

Supplement: Supplementary file 6 — Figure S6. [file CAM4-13-e6736-s016.tif]

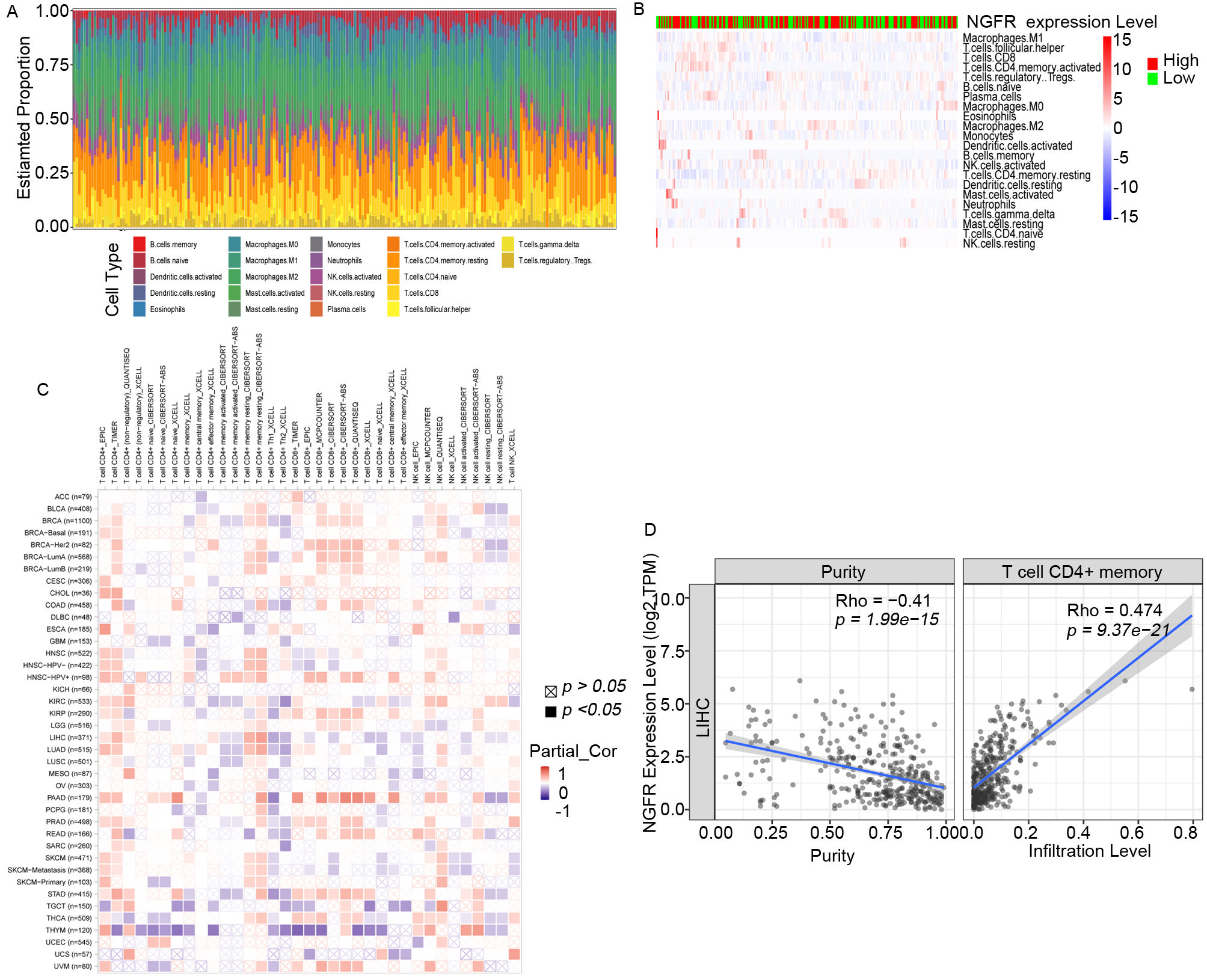

Supplement: Supplementary file 7 — Figure S7. [file CAM4-13-e6736-s001.tif]

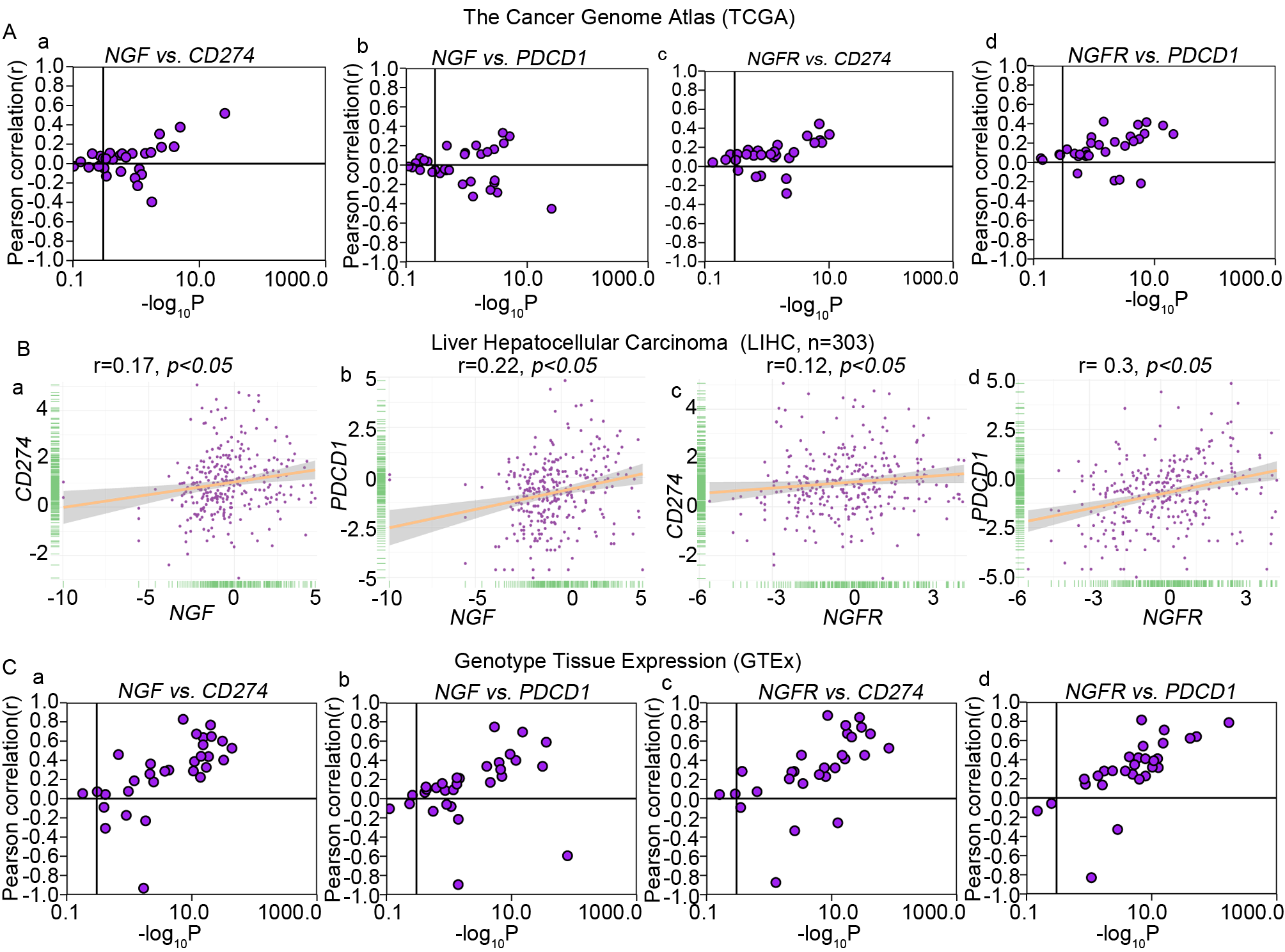

Supplement: Supplementary file 8 — Figure S8. [file CAM4-13-e6736-s007.tif]

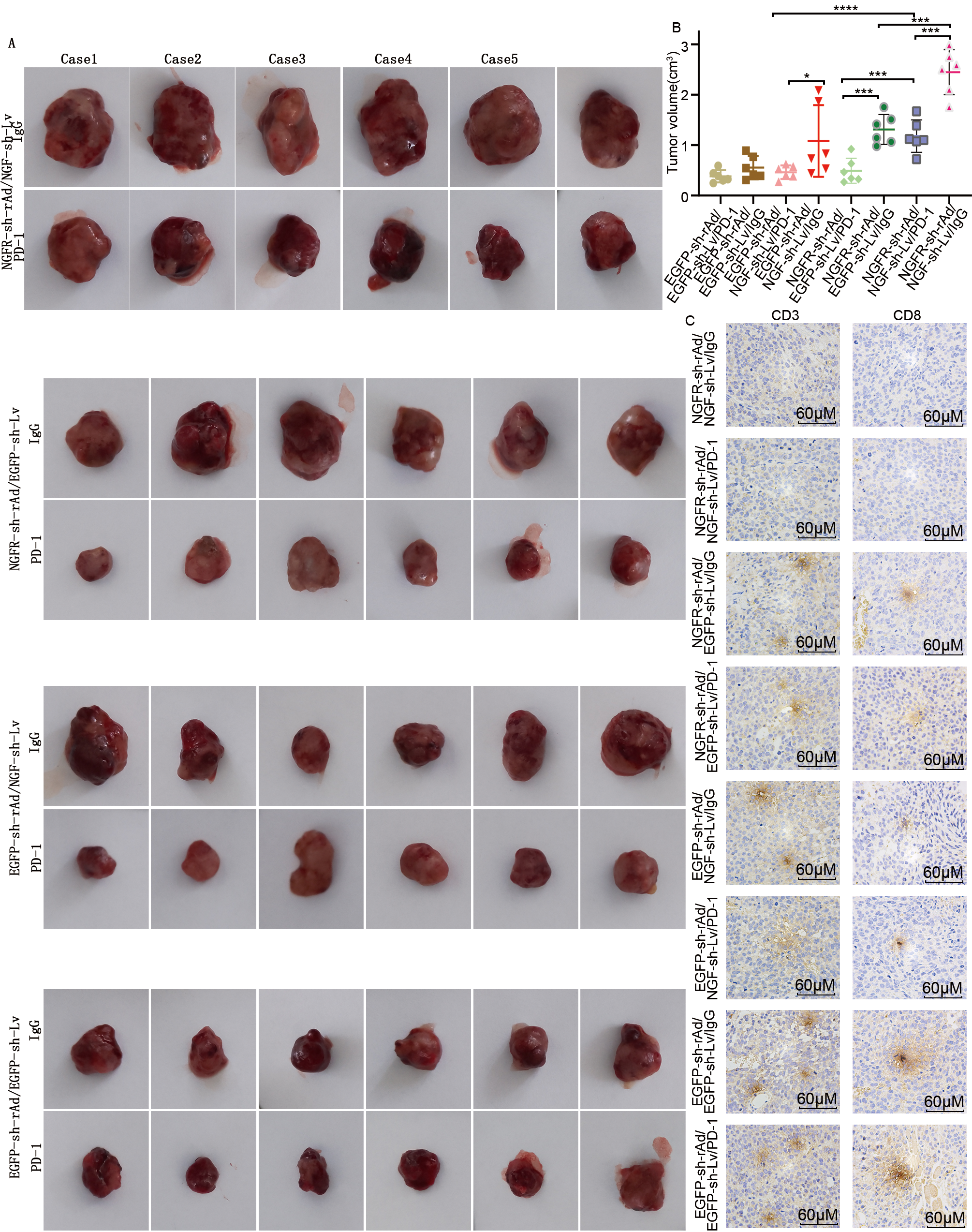

Supplement: Supplementary file 9 — Figure S9. [file CAM4-13-e6736-s015.tif]

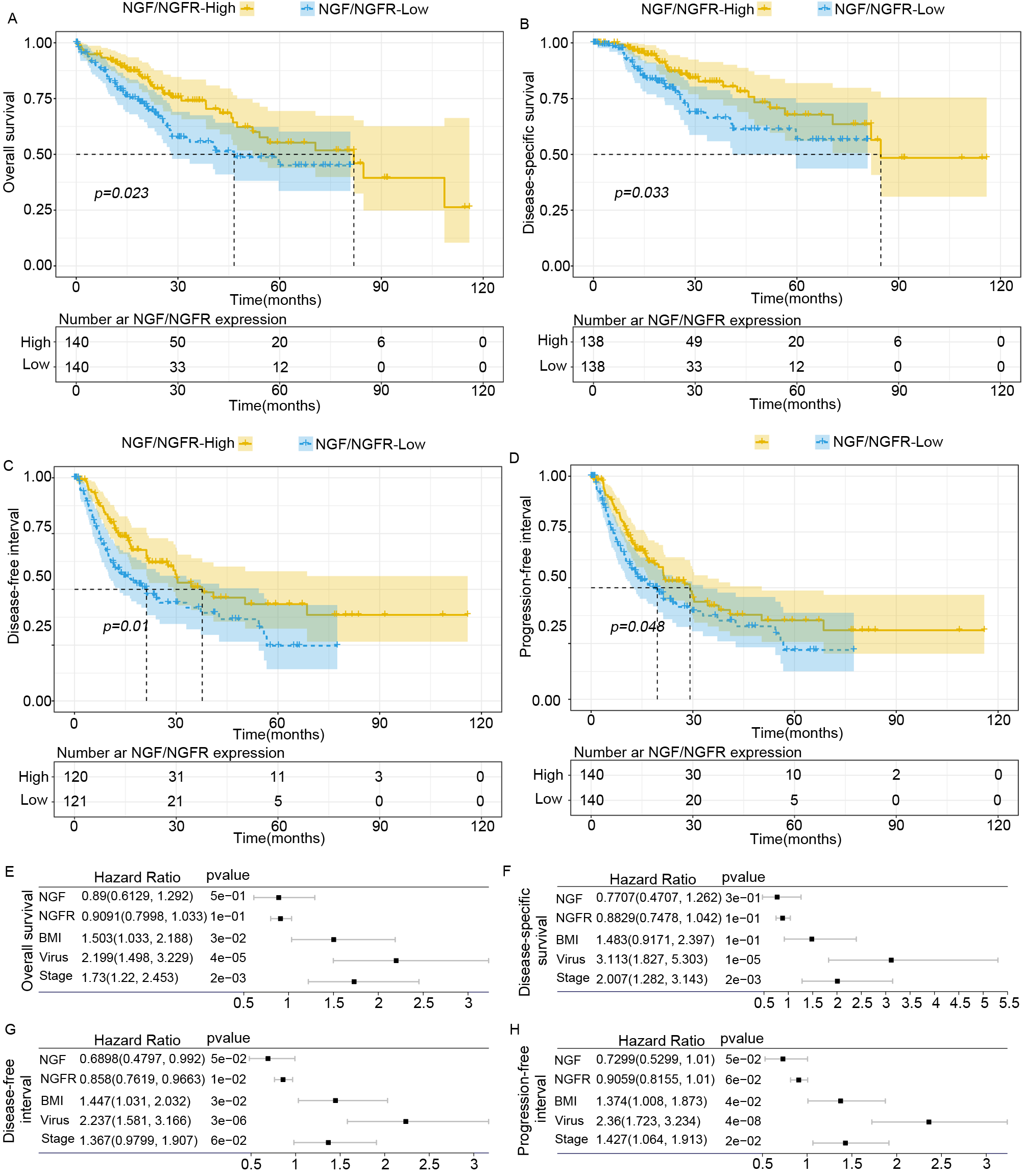

Supplement: Supplementary file 10 — Figure S10. [file CAM4-13-e6736-s008.tif]

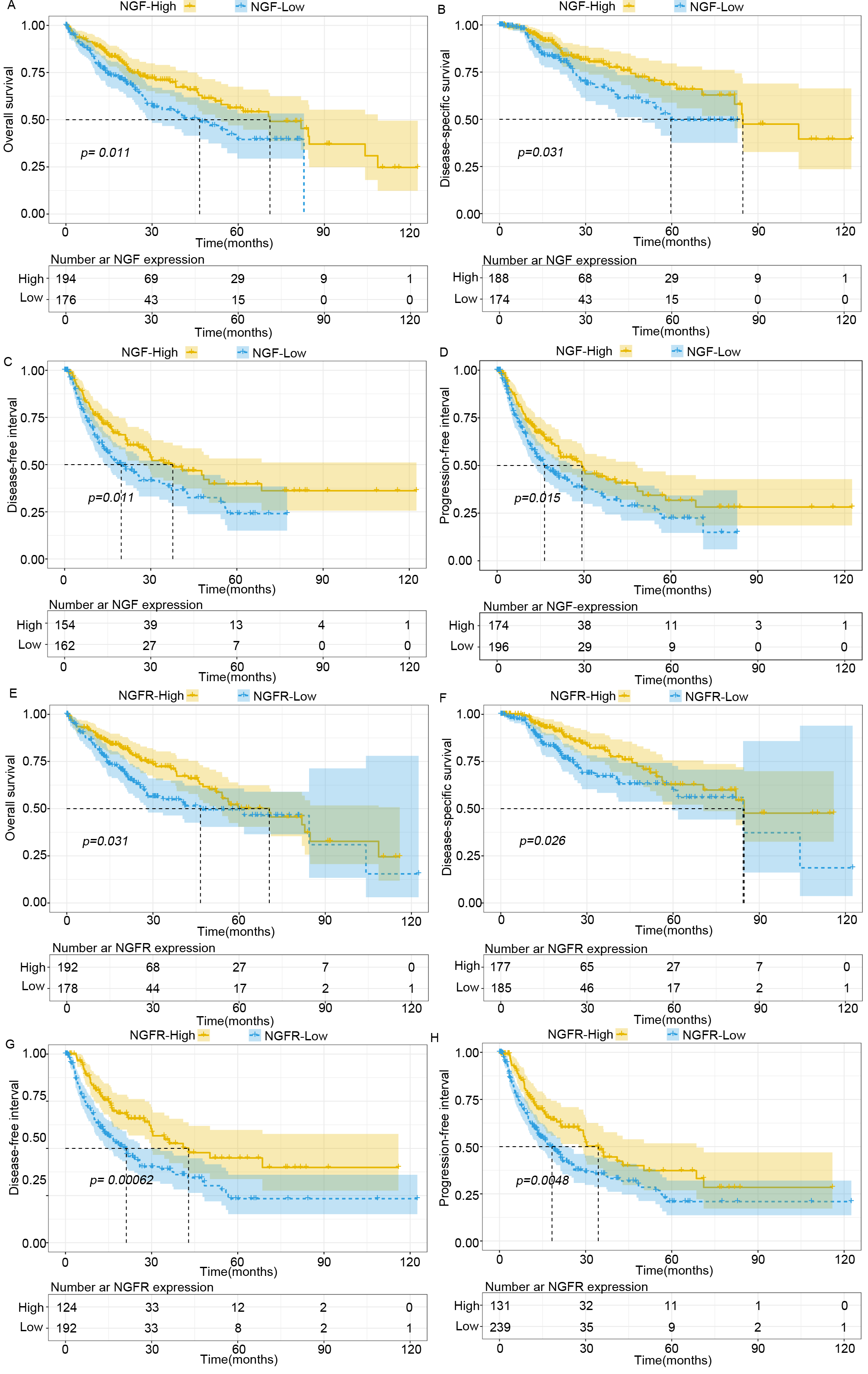

Supplement: Supplementary file 11 — Figure S11. [file CAM4-13-e6736-s009.tif]
