# Supplementary material for: Heterogeneity‐induced NGF‐NGFR communication inefficiency promotes mitotic spindle disorganization in exhausted T cells through PREX1 suppression to impair the anti‐tumor immunotherapy with PD‐1 mAb in hepatocellular carcinoma
Source: Cancer Med. 2024 Jan 10;13(3):e6736. doi: 10.1002/cam4.6736 (PMC10905245; doi:10.1002/cam4.6736)
Supplement: Supplementary file 12 — Table S1. [file CAM4-13-e6736-s005.docx]

| Table S1. Clinicopathologic characteristics of HCC patients | | | | | | | | | |
| --- | --- | --- | --- | --- | --- | --- | --- | --- | --- |
| Sex | Age | AFP | MELD | CHILDL | HBV | Numbers | Size | Invasion | TNM |
| Male | 75 | 2202 | 18 | 6 | No | Multiple | 8.6 | Yes | 4 |
| Male | 70 | 42.45 | 35 | 8 | Yes | Multiple | 2.5 | No | 3 |
| Female | 69 | 44.86 | 11 | 7 | Yes | Single | 2.5 | No | 2 |
| Male | 69 | 13.72 | 7 | 6 | Yes | Single | 6.5 | No | 3 |
| Male | 68 | 1210 | 40 | 11 | No | Multiple | 3.6 | No | 2 |
| Male | 68 | 16.86 | 8 | 8 | No | -1 | -1 | -1 | -1 |
| Male | 67 | 3.11 | 21 | 8 | Yes | Single | 5 | No | 1 |
| Male | 66 | 12.14 | 31 | 7 | Yes | Multiple | 2.5 | No | 2 |
| Male | 66 | 1500 | 31 | 8 | Yes | Multiple | 3 | Yes | 4 |
| Male | 65 | -2 | 10 | 9 | No | Single | 12 | Yes | 1 |
| Male | 64 | 23.66 | 7 | 5 | Yes | Multiple | 11 | No | 1 |
| Male | 64 | 10.63 | 36 | 9 | Yes | Multiple | 7 | No | 4 |
| Male | 63 | 498.9 | 35 | 9 | Yes | Multiple | 2 | No | 4 |
| Male | 63 | 0.87 | 10 | 5 | Yes | Single | 17 | No | 3 |
| Male | 62 | 321 | 8 | 5 | Yes | Multiple | 2.5 | No | 2 |
| Male | 62 | 43.7 | 7 | 5 | Yes | Single | 4 | No | 2 |
| Female | 62 | 25.23 | 35 | 8 | Yes | Single | 7.4 | No | 1 |
| Female | 62 | 72.66 | 7 | 5 | Yes | -1 | -1 | -1 | -1 |
| Female | 61 | 867 | 9 | 6 | Yes | Multiple | 6.1 | No | 3 |
| Male | 61 | 8976 | 10 | 8 | Yes | Multiple | 3.3 | No | 3 |
| Male | 61 | 40.37 | 15 | 9 | Yes | Single | 18 | Yes | 2 |
| Male | 61 | 2.35 | 12 | 8 | Yes | Single | 2.5 | No | 2 |
| Male | 61 | 18.21 | 8 | 6 | Yes | Single | 3.5 | No | 1 |
| Male | 61 | 513.4 | 22 | 9 | No | Single | 2.5 | No | 2 |
| Male | 60 | 1.85 | 30 | 7 | Yes | Multiple | 6 | Yes | 3 |
| Male | 60 | 389 | 23 | 8 | Yes | Single | 4.5 | No | 3 |
| Male | 59 | 10237 | 34 | 10 | Yes | Multiple | 4.6 | No | 3 |
| Male | 59 | 34.77 | 25 | 8 | No | Multiple | 8.5 | No | 3 |
| Male | 59 | 34.07 | 33 | 11 | Yes | Single | 7.8 | Yes | 3 |
| Male | 59 | -2 | 8 | 6 | Yes | Single | 21 | Yes | 2 |
| Female | 59 | 13542 | 13 | 7 | Yes | Single | 9.7 | No | 1 |
| Male | 59 | 1.42 | 9 | 7 | Yes | Single | 3.4 | No | 2 |
| Male | 59 | 41.7 | 12 | 6 | Yes | Single | 11 | No | 3 |
| Male | 59 | -2 | 15 | 10 | Yes | Single | 5.7 | No | 2 |
| Male | 58 | 283.7 | 32 | 7 | Yes | Multiple | 2.5 | Yes | 4 |
| Male | 58 | 980 | 28 | 8 | Yes | Multiple | 2.1 | Yes | 4 |
| Male | 57 | 2315 | 29 | 8 | Yes | Multiple | 6.5 | Yes | 4 |
| Male | 56 | -2 | 33 | 7 | Yes | Multiple | 2 | No | 2 |
| Male | 56 | 304.1 | 32 | 7 | Yes | Multiple | 1.9 | No | 2 |
| Male | 56 | 17710 | 31 | 7 | Yes | Single | 1.7 | Yes | 4 |
| Male | 55 | 2213 | 7 | 5 | Yes | Multiple | 3 | No | 3 |
| Male | 54 | 76.32 | 13 | 8 | Yes | Multiple | 3 | No | 2 |
| Male | 54 | 3.64 | 9 | 6 | Yes | Multiple | 2.4 | No | 2 |
| Male | 54 | 1.14 | 35 | 8 | Yes | Multiple | 2.3 | No | 4 |
| Male | 54 | 14.37 | 40 | 8 | Yes | Multiple | 4.7 | No | 1 |
| Male | 54 | -2 | 16 | 8 | Yes | Multiple | 8 | No | 2 |
| Male | 54 | 641.8 | 32 | 9 | Yes | Multiple | 12.2 | No | 3 |
| Male | 53 | 8.57 | 12 | 5 | Yes | Multiple | 7.5 | Yes | 3 |
| Male | 53 | 298.2 | 7 | 7 | Yes | Multiple | 15 | No | 4 |
| Male | 53 | 2.68 | 9 | 6 | Yes | Multiple | 2.6 | No | 3 |
| Male | 53 | 665.1 | 8 | 5 | Yes | Multiple | 3.1 | No | 2 |
| Male | 53 | 311.6 | 7 | 5 | Yes | Single | 3 | No | 1 |
| Male | 53 | 73.86 | 11 | 9 | Yes | Single | 4.3 | No | 3 |
| Male | 53 | 4.86 | 6 | 6 | Yes | Single | 4 | No | 3 |
| Male | 53 | 1500 | 32 | 9 | Yes | Single | 5 | No | 1 |
| Male | 52 | 30.96 | 22 | 12 | Yes | Multiple | 1.3 | No | 1 |
| Male | 52 | 0.89 | 13 | 9 | No | Multiple | 8.5 | No | 5 |
| Male | 52 | 291 | 8 | 6 | Yes | Single | 5.1 | No | 1 |
| Male | 51 | 362.9 | 19 | 9 | Yes | Multiple | 12 | No | 3 |
| Male | 51 | 1.47 | 11 | 6 | Yes | Single | 4.7 | No | 1 |
| Female | 50 | 4.22 | 6 | 8 | Yes | Single | 14 | Yes | 1 |
| Male | 49 | 2.02 | 11 | 6 | Yes | Multiple | 1.4 | No | 2 |
| Male | 48 | 214 | 32 | 7 | Yes | Multiple | 0.74 | Yes | 4 |
| Male | 48 | 14828 | 35 | 7 | Yes | Multiple | 2.1 | No | 3 |
| Male | 48 | 2.97 | 7 | 5 | Yes | Multiple | 1.4 | No | 3 |
| Male | 47 | 1308 | 13 | 8 | Yes | Multiple | 1.8 | No | 2 |
| Male | 47 | 872.2 | 7 | 5 | Yes | Single | 17 | Yes | 4 |
| Female | 46 | 64.57 | 16 | 8 | Yes | Multiple | 3.4 | No | 2 |
| Male | 46 | 366.6 | 8 | 5 | Yes | Multiple | 4.9 | No | 2 |
| Male | 46 | 7.52 | 15 | 6 | Yes | Multiple | 4 | No | 2 |
| Male | 46 | 1210 | 20 | 11 | Yes | Multiple | 9.6 | No | 2 |
| Male | 46 | 215.4 | 14 | 7 | Yes | Single | 6 | No | 2 |
| Male | 45 | 1500 | 32 | 8 | Yes | Multiple | 2.5 | No | 2 |
| Male | 45 | 5.58 | 13 | 8 | Yes | Multiple | 1.3 | No | 3 |
| Male | 45 | 2.26 | 37 | 9 | Yes | Single | 2.3 | Yes | 2 |
| Female | 44 | 2145 | 9 | 5 | Yes | Multiple | 4.5 | Yes | 3 |
| Male | 44 | 554.7 | 15 | 10 | Yes | Multiple | 2.9 | No | 1 |
| Male | 44 | 1210 | 15 | 10 | Yes | Multiple | 20 | No | 2 |
| Female | 44 | 112.4 | 15 | 8 | Yes | Single | 4 | No | 2 |
| Female | 44 | 2907 | 11 | 6 | Yes | Single | 4.5 | No | 2 |
| Male | 44 | 10.92 | 9 | 6 | Yes | Single | 2.5 | No | 1 |
| Male | 44 | 358.7 | 11 | 6 | Yes | -1 | -1 | -1 | -1 |
| Male | 43 | 1058 | 40 | 10 | Yes | Multiple | 7.4 | No | 2 |
| Male | 42 | 1.89 | 32 | 9 | Yes | Multiple | 12 | No | 3 |
| Female | 42 | 2200 | 7 | 5 | Yes | Multiple | 4.7 | No | 3 |
| Male | 42 | 117.4 | 33 | 8 | Yes | Multiple | 1.4 | No | 2 |
| Male | 42 | 11.73 | 38 | 9 | Yes | Single | 3.4 | Yes | 4 |
| Male | 42 | 9.51 | 31 | 8 | Yes | Single | 2.4 | No | 1 |
| Male | 40 | 1335660 | 36 | 5 | Yes | Multiple | 12.6 | Yes | 3 |
| Male | 40 | 7.4 | 20 | 10 | Yes | Multiple | 11.5 | No | 2 |
| Male | 40 | 349.3 | 6 | 5 | Yes | Single | 8 | No | 3 |
| Male | 39 | 231.5 | 10 | 8 | Yes | Multiple | 3.8 | No | 4 |
| Male | 39 | 80 | 20 | 8 | Yes | Single | 2.5 | No | 2 |
| Male | 33 | 1500 | 6 | 6 | Yes | Single | 7 | No | 3 |
| Male | 31 | 2.97 | 10 | 5 | Yes | Single | 2.8 | No | 1 |
| Female | 30 | 1210 | 30 | 7 | Yes | Multiple | 10.8 | No | 3 |
| Male | 27 | 1200 | 34 | 7 | Yes | Multiple | 4.3 | No | 3 |
| **Notes:** AFP, Alpha-Fetoprotein; MELD, Model for end-stage liver disease; TNM, Tumor Node Metastasis | | | | | | | | | |
