# Supplementary material for: Heterogeneity‐induced NGF‐NGFR communication inefficiency promotes mitotic spindle disorganization in exhausted T cells through PREX1 suppression to impair the anti‐tumor immunotherapy with PD‐1 mAb in hepatocellular carcinoma
Source: Cancer Med. 2024 Jan 10;13(3):e6736. doi: 10.1002/cam4.6736 (PMC10905245; doi:10.1002/cam4.6736)
Supplement: Supplementary file 13 — Table S2. [file CAM4-13-e6736-s003.docx]

| Table S2. Clinicopathologic characteristics of PD-1 mAb therapy cohorts | | | | | | | | |
| --- | --- | --- | --- | --- | --- | --- | --- | --- |
| Sex | Age | Size | Numbers | Infiltration | Intratumoral thrombus | AFP(ug.L) | TNM stage | Child stage |
| Man | 46 | 12 | Single | Yes | M1 | 26238 | ⅢB | 7 |
| Man | 62 | 3.2 | Single | No | M1 | 1837 | Ⅱ | 5 |
| Man | 74 | 3.2 | Single | Yes | M2 | 72.3 | ⅢB | 6 |
| Man | 41 | 15 | Single | No | M2 | 16828 | ⅢA | 7 |
| Man | 69 | 6.4 | Single | No | M2 | 3.6 | Ⅱ | 6 |
| Man | 53 | 7 | Multiple | No | M2 | 30678 | ⅢA | 6 |
| Man | 58 | 3.5 | Multiple | Yes | M2 | 14143 | ⅢB | 7 |
| Female | 71 | 6.4 | Single | Yes | M0 | 7635 | ⅢB | 7 |
| Man | 42 | 11 | Single | No | M2 | 42664 | Ⅱ | 6 |
| Female | 67 | 3.8 | Single | No | M2 | 772 | Ⅱ | 7 |
| Man | 52 | 15 | Multiple | Yes | M2 | 8620 | ⅢB | 7 |
| Man | 50 | 3.3 | Multiple | No | M1 | 75.6 | Ⅱ | 5 |
| Man | 71 | 7.3 | Single | No | M0 | 2.83 | ⅠB | 5 |
| Man | 50 | 7.5 | Single | No | M0 | 23.76 | ⅠB | 6 |
| Man | 66 | 3 | Single | No | M0 | 10.41 | ⅠB | 5 |
| Man | 66 | 11 | Single | No | M1 | 12.72 | Ⅱ | 6 |
| Female | 78 | 9 | Multiple | No | M2 | 31.85 | ⅢA | 6 |
| Man | 66 | 1.5 | Single | No | M0 | 6.59 | ⅠA | 6 |
| Female | 74 | 9 | Single | Yes | M0 | 223 | ⅢA | 7 |
| Man | 32 | 5 | Multiple | No | M1 | 21830 | ⅢA | 5 |
| Man | 65 | 11 | Single | No | M2 | 215 | ⅢA | 5 |
| Man | 43 | 2.5 | Single | Yes | M2 | 132 | ⅠA | 5 |
| Man | 50 | 3.3 | Single | No | M1 | 56.62 | Ⅱ | 5 |
| Man | 49 | 6 | Single | Yes | M0 | 3.29 | ⅢB | 5 |
| Man | 67 | 5.2 | Multiple | No | M2 | 30369 | ⅢB | 5 |
| Female | 75 | 9 | Multiple | No | M1 | 31.85 | Ⅱ | 5 |
| Man | 64 | 8 | Single | No | M2 | 54.5 | ⅠA | 6 |
| Man | 41 | 1.8 | Multiple | No | M0 | 122.9 | ⅢB | 7 |
| Female | 70 | 12 | Single | No | M2 | 18.76 | ⅢB | 5 |
| Notes: BCLC, Barcelona Clinic Liver Cancer; AFP, Alpha-Fetoprotein | | | | | | | | |
