# Supplementary material for: Heterogeneity‐induced NGF‐NGFR communication inefficiency promotes mitotic spindle disorganization in exhausted T cells through PREX1 suppression to impair the anti‐tumor immunotherapy with PD‐1 mAb in hepatocellular carcinoma
Source: Cancer Med. 2024 Jan 10;13(3):e6736. doi: 10.1002/cam4.6736 (PMC10905245; doi:10.1002/cam4.6736)
Supplement: Supplementary file 14 — Table S3. [file CAM4-13-e6736-s010.docx]

| Table S3. Primers, shRNA and SiRNA sequences | | |
| --- | --- | --- |
| Primers |  |  |
| Gene Symbol | Forward Primer | Reverse Primer |
| Human NGF | Ctccggcacagcagagag | Tacgctatgcacctcagtgt |
| Human NGFR | Cctacggctactaccaggatg | Cacacggtgttctgcttgt |
| Human GAPDH | Atgaatgggcagccgttagg | Cccaatacgaccaaatcagagaat |
| shRNA sequences |  |  |
| Target Gene | Sense | Antisense |
| Human NGF-shRNA | Acaguguauucaaacaguatt | Uacuguuugaauacacugutt |
| Human NGFR-shRNA | Actgtagtaaatggcaatt | Uugccauuuacuacagutt |
| SiRNA sequences |  |  |
| Target Gene | Sense | Antisense |
| PREX1-SiRAN1 | Gcuccuagaaauuggugaaau | Auuucaccaauuucuaggauc |
| PREX1-SiRAN2 | Ggugatcaaagaccgugauua | Uaaucacggucuuugaucacc |
| PREX1-SiRAN3 | Ccuaugaaccacagcuuacaa | Uuguaagcugugguucauagg |
