# Supplementary material for: Heterogeneity‐induced NGF‐NGFR communication inefficiency promotes mitotic spindle disorganization in exhausted T cells through PREX1 suppression to impair the anti‐tumor immunotherapy with PD‐1 mAb in hepatocellular carcinoma
Source: Cancer Med. 2024 Jan 10;13(3):e6736. doi: 10.1002/cam4.6736 (PMC10905245; doi:10.1002/cam4.6736)
Supplement: Supplementary file 16 — Table S5. [file CAM4-13-e6736-s012.docx]

| Table S5. Mitotic spindle pathway in NGF/NGFR differential expressed patients | | | | | |
| --- | --- | --- | --- | --- | --- |
| Genes | logFC | AveExpr | t | P.Value | adj.P.Val |
| ARHGEF3 | -1.4472 | 1.3624 | -9.4673 | 1.41E-18 | 4.39E-17 |
| ARAP3 | -1.3679 | 0.8900 | -12.667 | 3.02E-29 | 2.31E-27 |
| ABR | -1.3662 | 1.4654 | -10.041 | 2.12E-20 | 7.64E-19 |
| SHROOM2 | -1.3006 | 0.4059 | -5.7720 | 2.12E-08 | 2.38E-07 |
| FSCN1 | -1.2855 | 3.2775 | -10.2813 | 3.56E-21 | 1.36E-19 |
| NCK2 | -1.2803 | 2.6769 | -7.1522 | 7.83E-12 | 1.33E-10 |
| FLNA | -1.2738 | 6.1593 | -9.5191 | 9.69E-19 | 3.05E-17 |
| KIF3C | -1.1877 | -0.8217 | -6.5693 | 2.54E-10 | 3.63E-09 |
| PREX1 | -1.0756 | 2.0875 | -9.1105 | 1.80E-17 | 5.13E-16 |
| RAPGEF5 | -1.0756 | 1.8367 | -7.9196 | 6.02E-14 | 1.27E-12 |
| CLIP2 | -0.9703 | 1.1206 | -5.0681 | 7.41E-07 | 6.42E-06 |
| DOCK2 | -0.9349 | 0.4935 | -6.0349 | 5.16E-09 | 6.24E-08 |
| OPHN1 | -0.9341 | -2.5173 | -4.3970 | 1.57E-05 | 0.0001 |
| ARHGAP27 | -0.8844 | 1.5748 | -6.6041 | 2.08E-10 | 2.99E-09 |
| SYNPO | -0.8832 | 4.1396 | -8.2920 | 5.10E-15 | 1.17E-13 |
| ARHGAP10 | -0.8380 | 0.8013 | -5.2058 | 3.80E-07 | 3.46E-06 |
| VCL | -0.8167 | 2.8494 | -7.3787 | 1.92E-12 | 3.49E-11 |
| FGD6 | -0.8038 | 0.1335 | -5.2526 | 3.02E-07 | 2.78E-06 |
| RASAL2 | -0.7790 | 0.2320 | -4.6513 | 5.14E-06 | 3.76E-05 |
| TIAM1 | -0.7736 | -0.5308 | -4.4451 | 1.28E-05 | 8.68E-05 |
| PKD2 | -0.7500 | 1.0185 | -4.2631 | 2.78E-05 | 0.0001 |
| HOOK3 | -0.7381 | 0.9375 | -4.9962 | 1.04E-06 | 8.81E-06 |
| GSN | -0.7328 | 6.3515 | -5.6293 | 4.48E-08 | 4.74E-07 |
| STK38L | -0.7276 | 1.3745 | -6.2921 | 1.24E-09 | 1.63E-08 |
| MYO1E | -0.7032 | 4.3495 | -5.6427 | 4.18E-08 | 4.45E-07 |
| MYH10 | -0.683 | 2.3238 | -4.3490 | 1.93E-05 | 0.0001 |
| PALLD | -0.6524 | 3.4340 | -3.7845 | 0.0001 | 0.0009 |
| FGD4 | -0.6368 | 0.9502 | -4.1477 | 4.49E-05 | 0.0002 |
| SORBS2 | -0.6356 | 4.4923 | -3.7724 | 0.0001 | 0.0010 |
| EPB41L2 | -0.6040 | 2.7011 | -4.2924 | 2.46E-05 | 0.0001 |
| MYH9 | -0.5922 | 6.4812 | -5.4364 | 1.21E-07 | 1.19E-06 |
| ARHGAP29 | -0.591 | 2.2916 | -4.4397 | 1.31E-05 | 8.87E-05 |
| EZR | -0.5602 | 4.4067 | -4.1734 | 4.04E-05 | 0.0002 |
| RHOF | -0.5246 | 1.1804 | -2.5171 | 0.0124 | 0.0352 |
| NOTCH2 | -0.5209 | 2.3108 | -3.4808 | 0.0005 | 0.0026 |
| MARCKS | -0.5158 | 3.9314 | -3.6055 | 0.0003 | 0.0017 |
| TOP2A | 0.58134 | 3.1627 | 2.8982 | 0.0040 | 0.0138 |
| BIRC5 | 0.66134 | 2.5805 | 3.4849 | 0.0005 | 0.0025 |
