# Supplementary material for: Heterogeneity‐induced NGF‐NGFR communication inefficiency promotes mitotic spindle disorganization in exhausted T cells through PREX1 suppression to impair the anti‐tumor immunotherapy with PD‐1 mAb in hepatocellular carcinoma
Source: Cancer Med. 2024 Jan 10;13(3):e6736. doi: 10.1002/cam4.6736 (PMC10905245; doi:10.1002/cam4.6736)
Supplement: Supplementary file 17 — Data S1. [file CAM4-13-e6736-s004.docx]

### 1. Supplementary Methods:

### 1.1. Cell culture

Jurkat T (RRID:CVCL_0367) and Huh7 (RRID:CVCL_B7TI) cells were purchased from and authorized by the China Center for Type Culture Collection (CCTCC). The two types of cells were cultured in RPMI-1640 or DMEM supplemented with 100 U/mL penicillin, 100 μg/mL streptomycin, and 10% fetal bovine serum. The PBMCs separated CD3^+^ T cells were cultured in RPMI-1640 supplemented with 100 U/mL penicillin 100, μg/mL streptomycin, and 10% fetal bovine serum. Recombinant Human IL-2 was added for the proliferation of CD3^+^ cells.

### 1.2. CD3^+^ T cell separation and culture

The lymphocyte separation medium from Solarbio Life Sciences was used for the separation of PBMCs from freshly collected whole blood cells, and the residual red blood cells were removed using the red blood cell lysis buffer obtained from Solarbio Life Sciences. The CD3/CD28 conjugated magnetic beads were used for separating CD3^+^ T cells from the PBMCs. Afterward, the CD3/CD28 magnetic beads-conjugated CD3^+^T cells were cultured in the Human IL-2 enriched RPMI-1640 medium for two weeks.

### 1.3. Vectors

HEK-293A cells were double-transfected with pDC316-NGFR-shRNA and pBHGlox-E1,3Cre using Lipofectamine 3 000 to produce the NGFR-shRNA-rAd vector. HEK-293T cells were triple-transfected with pLV-NGF-shRNA and two packaging plasmids, pH1 and pH2 using Lipofectamine 3 000 to construct the NGF-shRNA-Lv vector. The interfering sequences of the target genes NGF and NGFR are listed in Table S2.

### 1.4. Xenograft HCC mouse model

Huh7 cells (2×10^6^ in 100 μl) and an equal volume of Matrigel were mixed and then injected subcutaneously into immune-deficient mice for the establishment of the mouse model. The immune system was reconstituted by injecting CD3^+^ T cells into the tail vein of the mice, followed by therapy with PD1 mAb or an IgG. The tumor sizes were measured every week using vernier calipers. Finally, a paraffin section was constructed for the immunohistochemistry staining of CD8 and CD3.

### 1.5. EdU assay

The EdU assay was conducted in the co-culture system following the manufacturer's protocol. Briefly, the cells were exposed to 10μM of EdU for one hour. Jurkat T cells were collected and treated with 70% ethanol for 15 min for fixing. The cell membrane system was penetrated with 0.1% Triton-X 100 solution, followed by incubation with Azide Alexa Fluor 488. Next, the DAPI solution was added for the staining of genomic DNA. A fluorescence microscope was employed to visualize the positive EdU cells.

### 1.6. Flow cytometry assay

Jurkat T cell pellet was collected from the co-culture system and treated with 70% ethanol for 15 min for fixing. The RNA in the cells was digested using RNase, and the genomic DNA was stained with 1 mg/ml of PI for 30 min. After filtering through a 400-mesh sieve, the PI fluorescence intensity of the genomic DNA was recorded using a flow cytometer (FACSCalibur). Afterward, the cell cycle distribution was determined.

### 1.7. Immunohistochemistry assay

Tissue sections were treated with xylene for deparaffination followed by dehydration using a gradient alcohol. In a pressure cooker, tissue sections were mixed with an antigen retrieval solution, and the endogenous peroxidase was blocked using hydrogen peroxide. The tissue section was then incubated with the primary antibodies for NGF, NGFR, CD3, and CD8 at 37°C for 60 min followed by labeling with biotin-labeled goat anti-rabbit/mice IgG. After another incubation with horseradish enzyme-labeled streptavidin for 30 min, freshly prepared DAB solution was added to the tissue section. Next, hematoxylin staining was performed, followed by another round of xylene treatment and dehydration using gradient alcohol. A microscope was employed to visualize the expressions of NGF, NGFR, CD3, and CD8 in the tissues.

1.8. Immunoblot assay

The protein in the tissues or cell pellets was extracted using RIPA buffer and then quantified using the BCA Protein Quantitation Kit from Solarbio Life Sciences. After being separated on SDS-PAGE gels, the protein was transferred to PVDF membranes, which were then incubated with the primary antibody (NGF, NGFR, α-tubulin, PREX1, HDAC1, tubulin, and β-actin) for the individual antigen. Specific antigen-conjugated primary antibody was then recognized using the HRP-linked secondary antibody. Tanon imaging system was employed for detecting the target bands after incubation with the electrochemiluminescence solution for two mins.

1.9. Separation of monomeric and polymeric tubulin

The monomeric tubulin in Jurkat T cells was extracted using the monomeric extraction buffer, as described by T.L. [1]. The monomeric fraction of tubulin in the NP-40 solution was collected after 10 min of centrifugation at 13, 000g. Subsequently, the polymeric fraction of tubulin in the insoluble pellet was resuspended in RIPA buffer, followed by 15 min of centrifugation at 13,000 g. After boiling in the SDS loading buffer for 5 minutes, an equivalent amount of total protein was separated on a 12% SDS-PAGE gel.

1.10. Nuclear extraction

The unclear fraction of Jurkat T cells was separated as described in previous report [2]. Briefly, the cell pellet was collected from the co-culture system, resuspended in the pre-extraction buffer, and centrifuged at 8,000g. The obtained cytoplasmic fraction was transferred to a fresh EP tube. Subsequently, the insoluble nuclear pellet was resuspended in the extraction buffer and sonicated in an ultrasonic disintegrator. The nuclear fraction was collected after centrifugation at 12,000g.

1.11. Immunofluorescence staining

Jurkat T cells were collected from the co-culture system and resuspended in 75% alcohol for 15 min. Triton X-100 was used for the penetration of the membrane system. After three washes with PBS, the cell suspension was incubated with primary antibodies (α-tubulin, Lamin B, HDAC1, and H3-ser28). After another three washes with PBS, secondary antibody, FITC, or TRITC connected and diluted at 1:400 was added, followed by incubation. Subsequently, the DNA content in the Jurkat T cells was stained using a DAPI solution. A fluorescence microscope was employed to observe the subcellular localization of α-tubulin, Lamin B, and HDAC1.

### 1.12. Quantitative real-time polymerase chain reaction

The tissue sample (100 mg) was homogenized in TRIzol solution for total RNA isolation as described in a previous report [3]. The extracted RNA (500 ng/µl) was stored in RNase-free water. SuperScript® III, a First-Strand Synthesis kit, was utilized to synthesize the cDNA as described previously[4]. Sybr Green reagent was used for the quantitative real-time polymerase chain reaction of the synthesized cDNA in the PCR machine by Applied Biosystems. The relative expressions of NGF and NGFR in HCC patients were determined using the -∆∆Cq method. The primer pairs were synthesized in Sangon Biotech and are presented in Table S3.

**Supplementary References:**

1. Lee ST, Lee JY, Han CR, et al. Dependency of 2-methoxyestradiol-induced mitochondrial apoptosis on mitotic spindle network impairment and prometaphase arrest in human Jurkat T cells. Biochem Pharmacol. 2015; 94(4):257-69. doi: 10.1016/j.bcp.2015.02.011.

2. Gao Y, Xu Q, Li X, et al. Heterogeneity induced GZMA-F2R communication inefficient impairs antitumor immunotherapy of PD-1 mAb through JAK2/STAT1 signal suppression in hepatocellular carcinoma. Cell Death Dis. 2022; 13(3):213. doi: 10.1038/s41419-022-04654-7.

3. Yang T, Zhu C, Shi Y, et al. RDIVpSGP motif of ASPP2 binds to 14-3-3 and enhances ASPP2/k18/14-3-3 ternary complex formulation to promote BRAF/MEK/ERK signal inhibited cell proliferation in hepatocellular carcinoma. Cancer Gene Ther. 2022; 29(11):1616-1627. doi: 10.1038/s41417-022-00474-1.

4. Yang T, Ouyang Y, Gao Y, Liu D, Zang Y, Chen D. Enriched high‑throughput reverse transcription‑quantitative PCR template preparation without pre‑amplification. Mol Med Rep. 2020; 22(4):3541-3548. doi: 10.3892/mmr.2020.11389.

**2. Supplementary Figure legends**

**Supplementary Figure 1**. Landscape of the NGF-enriched GSEA pathway in pan-cancer analysis (33 cancer types) and LIHC. (A) GSEA depicting the pathway of NGF in the pan-cancer analysis. (B) The NGF-activated/suppressed pathway ratio in the pan-cancer analysis. (C) The NGF-activated hallmarks of apoptosis in LIHC and pan-cancer analysis. Ca The NGF-activated hallmarks of apoptosis in LIHC, Cb the correlation of NGF expression and hallmarks of apoptosis in LIHC and Cc the correlation of NGF expression and hallmarks of apoptosis in pan-cancer. (D) The NGF-activated hallmarks of mitotic spindle formation in LIHC and pan-cancer analysis. Da The NGF-activated hallmarks of mitotic spindle formation in LIHC, Db the correlation of NGF expression and hallmarks of mitotic spindle formation in LIHC and Dc the correlation of NGF expression and hallmarks of mitotic spindle formation in pan-cancer.

**Supplementary Figure 2**. Landscape of the NGFR-enriched GSEA pathway in pan-cancer analysis (33 cancer types) and LIHC. (A) GSEA presenting the pathway of NGFR in the pan-cancer analysis. (B) The NGFR activated/suppressed pathway ratio in the pan-cancer analysis. (C) The NGFR-activated hallmarks of apoptosis in LIHC and pan-cancer analysis. Ca The NGFR-activated hallmarks of apoptosis in LIHC, Cb the correlation of NGFR expression and hallmarks of apoptosis in LIHC and Cc the correlation of NGFR expression and hallmarks of apoptosis in pan-cancer. (D) The NGFR-activated hallmarks of mitotic spindle formation in LIHC and pan-cancer analysis. Da The NGFR-activated hallmarks of mitotic spindle formation in LIHC, Db the correlation of NGFR expression and hallmarks of mitotic spindle formation in LIHC and Dc the correlation of NGFR expression and hallmarks of mitotic spindle formation in pan-cancer.

**Supplementary Figure 3**. The NGF-NGFR communication inefficiency regulated singles. (A) Expression heatmap of the differentially expressed genes between NGF/NGFR low expression and NGF/NGFR high expression groups of tumors. (B) Volcano plot for the differentially expressed genes between NGF/NGFR low-expression and NGF/NGFR high-expression groups of tumors. (C) Gene enrichment analysis revealing the molecular function of the differentially expressed genes between NGF/NGFR low expression and NGF/NGFR high-expression groups of tumors. (D) Gene enrichment analysis revealing the biological processes of the differentially expressed genes between the NGF/NGFR low-expression and NGF/NGFR high-expression groups of tumors. (E) Gene enrichment analysis revealing the cell composition related to the differentially expressed genes between the NGF/NGFR low-expression and NGF/NGFR high-expression groups of tumors. (F) Gene Set Enrichment Analysis of the differentially expressed genes between the NGF/NGFR low-expression and NGF/NGFR high-expression groups of tumors.

**Supplementary Figure 4**. NGF-NGFR communication inefficient in the antitumor immunotherapy of PD-1 mAb. (A-B) Tumors of Huh7 cell infected with NGF-shRNA-Lv in immunodeficient mice tail vein injected with NGFR-shRNA-rAd infected CD3^+^ T cells. Immune system reconstituted immunodeficient mice were exposed to PD-1 mAb, subsequently. n=6, **p<0.05*, ***p<0.01*, ****p<0.001*, *****p<0.0001*. (C) CD3 and CD8 expression was recorded with immunohistochemistry staining in tumor tissues of mice. Scale bars, 60 μm. (D) The knockout efficiency of NGF and NGFR.

**Supplementary Figure 5**. Function role of NGF and NGFR expression in HCC patients. Clinical role of NGF expression of the overall (A), disease-specific (B), disease-free interval (C), and progression-free survival (D) in LIHC patients. Clinical role of NGFR expression of the overall (E), disease-specific (F), disease-free interval (G), and progression-free survival (H) in LIHC patients.

**Supplementary Figure 6**. The landscape of immune infiltration in the NGF high-expression and low-expression patients. (A) Bar plot illustrating immune infiltration in the NGF high-expression and low-expression LIHC patients (B) Heatmap illustrating immune infiltration in the NGF high-expression and low-expression LIHC patients. (C) Correlation of NGF expression with immune infiltration in pan-cancer analysis. (D) The positive correlation of NGF expression with infiltrated memory CD4^+^ T cells (Da), CD8^+^ T cells (Db) and activated NK cells (Dc) in LIHC.

**Supplementary Figure 7**. Landscape of immune infiltration in the NGFR high-expression and low-expression patients. (A) Bar plot illustrating immune infiltration in the NGFR high-expression and low-expression LIHC patients (B) Heatmap illustrating immune infiltration in the NGFR high-expression and low-expression LIHC patients. (C) Correlation of NGF expression with immune infiltration in pan-cancer analysis. (D) The positive correlation of NGF expression with infiltrated memory CD4^+^ T cell in LIHC.

**Supplementary Figure 8**. Co-expression of PD-1 and PD-L1 with NGF and NGFR. (A) Co-expression of NGF with PD-1 (Aa) and PD-L1 (Ab), and NGFR with PD-1 (Ac) and PD-L1 (Ad) in tumors from TCGA. (B) Co-expression of NGF with PD-1 (Ba) and PD-L1 (Bb), and NGFR with PD-1 (Bc) and PD-L1 (Bd) in tumors from LIHC. (C) Co-expression of NGF with PD-1 (Ca) and PD-L1 (Cb), and NGFR with PD-1 (Cc) and PD-L1 (Cd) in tumors from GTEx.

**Supplementary Figure 9**. Edu staining of PREX1 expression on NGF-NGFR communication inefficient. (A) Edu staining was performed for the effect of PREX1-siRNA on cell proliferation of Jurkat cells. (B) Edu staining was performed for the effect of PREX1 overexpression on cell proliferation of Jurkat cells. Scale bars, 60 μm. Genomic DNA was stained in blue with DAPI solution. N=10, **p<0.05*, ***p<0.01*, ****p<0.001*.

**Supplementary Figure 10**. Function role of NGF and NGFR expressions in HCC patients. Clinical role of NGF/NGFR expression in the (A) overall, (B) disease-specific, (C) disease-free interval, and (D) progression-free survival in LIHC patients. (E-H) Cox multivariate proportional hazard regression model for multivariate analysis of the hazard ratios (HRs); low expression of NGF or NGFR could serve as an independent prognostic factor for the (E) overall, (F) disease-specific, (G) disease-free interval, and (H) progression-free survival.

**Supplementary Figure 11**. H3-Ser28 staining of PREX1 expression on NGF-NGFR communication inefficient. (A) H3-Ser28 staining was performed for the effect of PREX1-siRNA on cell proliferation of Jurkat cells. (B) H3-Ser28 staining was performed for the effect of PREX1 overexpression on cell proliferation of Jurkat cells. Scale bars, 60 μm. Genomic DNA was stained in blue with DAPI solution. N=10, **p<0.05*, ***p<0.01*, ****p<0.001*.
